# New *MacrochelePratums* species (Acari, Mesostigmata, Macrochelidae) associated with burying beetles (Silphidae, *Nicrophorus*) in North America

**DOI:** 10.3897/zookeys.721.21747

**Published:** 2017-12-12

**Authors:** Wayne Knee

**Affiliations:** 1 Canadian National Collection of Insects, Arachnids, and Nematodes, Agriculture and Agri-Food Canada, 960 Carling Avenue, K.W. Neatby Building, Ottawa, Ontario, K1A 0C6, Canada

**Keywords:** Acari, carrion-feeding, COI, ecology, mite, phoresy

## Abstract

Burying beetles (Silphidae, *Nicrophorus*) are hosts to a broad diversity of mites (Acari), including several species of *Macrocheles* Latreille, 1829 (Mesostigmata, Macrochelidae). The macrochelid fauna associated with silphids primarily in North America was surveyed; in total, 1659 macrochelids representing seven species were collected from 112 *Nicrophorus* beetles representing nine host species. Three new species of *Macrocheles* were discovered during the survey and described as *Macrocheles willowae* **sp. n.**, *M. pratum* **sp. n.**, and *M. kaiju* **sp. n.** The barcode region of cytochrome oxidase subunit I (COI) was amplified from the three new described species, as well as *M. nataliae* and *M. praedafimetorum*, and analysed in a small phylogeny.

## Introduction

Carrion-feeding beetles (Silphidae) are associated with a diverse assemblage of mites, nematodes, and fungi. *Nicrophorus* (Silphidae) species are large-bodied beetles, that breed and feed on decaying organic matter, most often vertebrate carcasses (Anderson and Peck 1985). There are at least 60 extant species of *Nicrophorus* worldwide, 22 of which occur in the New World (Sikes et al. 2008, Sikes et al. 2016). *Nicrophorus* beetles are unique amongst insects because most species provide biparental care and they bury small vertebrate carcasses in subterranean crypts (see Anderson and Peck (1985) for a summary of their life cycle). *Nicrophorus* beetles are associated with a broad diversity of mites that can occur at high prevalences and abundances, with at least 14 species of mites representing four families collected off 95% of beetles in a given population (Wilson and Knollenberg 1987). The symbiotic relationship between silphids and their associated mites are poorly understood; however, the relationship may be a blend of commensalism and mutualism, as some mite species actively prey on eggs of carrion-feeding flies that compete with *Nicrophorus* (Wilson and Knollenberg 1987).

The Macrochelidae (Mesostigmata) are a cosmopolitan family of predaceous mites with at least 480 described species from 20 genera, occurring in a wide variety of organic substrates where they feed on nematodes and other microinvertebrates (Krantz 1998, Lindquist et al. 2009, Emberson 2010). There are about 320 described species of *Macrocheles* Latreille, 1829 (Macrochelidae) worldwide (Emberson 2010), many of which are phoretic as adult females on insects, including nine species which are associated with silphids (*M. agilis*, *M. glaber*, *M. kurosai*, *M. lisae*, *M. merdarius*, *M. muscaedomesticae*, *M. nataliae*, *M. praedafimetorum*, *M. vespillo*) (Halliday 2000, Mašán 2003, Niogret et al. 2007, Perotti and Braig 2009). *Macrocheles* associated with silphids attach with their chelicerae to beetles dispersing to and from carcasses, and they generally feed on nematodes, insect eggs and larvae, and other invertebrates on carrion (Wilson and Knollenberg 1987, Schwarz et al. 1998). Macrochelids phoretic on burying beetles are often overlooked and unstudied, resulting in a scarcity of information about their life history and novel species that remain to be described. A recent survey of tortoise mites (Uropodina, *Uroobovella*) on *Nicrophorus* beetles (Knee et al. 2012) also uncovered three new species of *Macrocheles* associated with burying beetles. Herein, I propose and describe *Macrocheles willowae* sp. n., *M. pratum* sp. n., and *M. kaiju* sp. n., include a small phylogeny based on the barcode region of COI, and describe the diversity, abundance and host range of *Macrocheles* species found on *Nicrophorus* throughout this survey.

## Methods

### Biological collections

Silphids were collected by various researchers across eight countries and 21 provinces or states (see acknowledgments). In Canada, most silphids were collected as bycatch from xylophagous beetle trapping by W.K. Specimens from other countries were collected primarily in pitfall traps, and others were hand-collected. Beetle specimens preserved in ethanol were shipped to Carleton University, and upon receipt specimens were placed in 95% ethanol and stored at -20°C. Using a dissecting microscope, silphids were identified to species using keys from Anderson and Peck (1985). The presence, abundance, and attachment location of mesostigmatic mites was recorded. All mesostigmatic mites were removed and placed in a 0.5 ml microfuge tube with 95% ethanol and stored at -20°C for later identification and/or molecular analysis. Mites were slide-mounted in polyvinyl alcohol medium (6371A, BioQuip Products, Rancho Dominguez, California, United States of America (USA)) and cured on a slide warmer at 40°C for 3–4 days. Slide-mounted specimens were examined using a compound microscope (Leica DM 2500) with differential interference contrast illumination (DIC), and identified to species using the primary literature. Initial drawings of mites were made with pencil on paper using a camera lucida. Illustrations were later merged in Adobe Photoshop CS5 and redrawn in Adobe Illustrator CS5 using an Intuos 3 Graphics Tablet from WACOM Co., Ltd. (Saitama, Japan). Leg chaetotaxy is based on the system proposed by Evans (1963) and Evans and Till (1965). Idiosomal chaetotaxy follows the system of Lindquist and Evans (1965) as applied to macrochelids by Hyatt and Emberson (1988). Notation for glandular openings and poroids (proprioreceptors or lyrifissures) follows the system developed by Athias-Henriot (1969, 1971, 1975) and Johnston and Moraza (1991), as reviewed by Kazemi et al. (2014). Measurements were made from at least eight female specimens, all measurements are in micrometres (µm), and lengths presented with mean followed by the range in parenthesis. Type specimens are deposited in the Canadian National Collection of Insects, Arachnids, and Nematodes (CNC), at Agriculture and Agri-Food Canada, Ottawa, Ontario, Canada, and the Smithsonian Institution National Museum of Natural History.

### Molecular methods

Genomic DNA was extracted from whole specimens for 24 hours using a DNeasy Tissue kit (Qiagen, Inc., Santa Clara, California, USA). Following extraction, mites were removed from the extraction buffer, vouchers were-slide mounted, and genomic DNA was purified following the DNeasy Tissue kit protocol. PCR amplifications were performed in a total volume of 25 µl, with 14.7 µl ddH2O, 2.5 µl 10× ExTaq buffer, 0.65 µl 25 mM MgCl2, 1.0 µl of each 10 µM primer, 2.0 µl 10 mM dNTPs, 0.15 µl ExTaq DNA polymerase, and 3 µl genomic DNA template. Primer pairs LCO1490 + HCO2198 (Folmer et al. 1994) were used to amplify a 689 bp fragment of the 5’–end of COI. PCR amplification was performed on an Eppendorf ep Gradient S Mastercycler (Eppendorf AG, Hamburg, Germany), using the following protocol: initial denaturation cycle at 94 °C for 3 min, followed by 45 cycles of 94 °C for 45 s, primer annealing at 45 °C for 45 s, 72 °C for 1 min, and a final extension at 72 °C for 5 min. Amplified products and negative controls were visualized on 1% agarose electrophoresis gels, and purified using pre-cast E-Gel CloneWell 0.8% SYBR Safe agarose gels (Invitrogen, Carlsbad, California, USA). Sequencing reactions followed the protocol of Knee et al. (2012), and sequencing was performed at the Agriculture and Agri-Food Canada, Eastern Cereal and Oilseed Research Centre Core Sequencing Facility (Ottawa, Ontario, Canada).

Sequence chromatograms were edited and contiguous sequences were assembled using Sequencher v5.3 (Gene Codes Corp., Ann Arbor, Michigan, USA). COI sequences were aligned manually in Mesquite v3.10 (Maddison and Maddison, 2016) according to the translated amino acid sequence. COI sequences from *Macrocheles subbadius* (MBIOE1677-13, MBIOE1699-13) generated by the Barcode of Life Data Systems (BOLD) were included in the phylogeny. COI sequences on GenBank from two Macronyssidae (Mesostigmata) species, *Ornithonyssus bacoti* and *O. sylviarum* (FM179677, KR103486), were used as outgroup sequences. Sequences generated during this study have been submitted to GenBank (Table 1).

**Table 1.** Collection information, host species records and mite abundance of *Macrocheles* (Macro.) mites collected from *Nicrophorus* (Nicro.) beetles, with GenBank accession numbers for COI.

Beetle number	Beetle species	Collection location	Coordinates	Collection date	*Macrocheles* species	Mite abundance	GenBank number
N002	*Nicro. defodiens*	CAN, ON, Algonquin P.P. 2	45.895, -78.071	16.vi.08	*Macro. willowae* sp. n.	1	–
N003	*Nicro. defodiens*	CAN, ON, Algonquin P.P. 2	45.895, -78.071	16.vi.08	*Macro. willowae* sp. n.	1	–
N005	*Nicro. orbicollis*	CAN, ON, Frontenac	44.447, -76.577	17.vi.08	*Macro. willowae* sp. n.	4	–
N006	*Nicro. orbicollis*	CAN, ON, Charleston Lake	44.500, -76.072	17.vi.08	*Macro. willowae* sp. n.	4	–
N007	*Nicro. orbicollis*	CAN, ON, Charleston Lake	44.500, -76.072	17.vi.08	*Macro. willowae* sp. n.	1	–
N008	*Nicro. orbicollis*	CAN, ON, Charleston Lake	44.500, -76.072	17.vi.08	*Macro. willowae* sp. n.	1	–
N011	*Nicro. defodiens*	CAN, ON, Algonquin P.P. 1	45.902, -77.605	16.vi.08	*Macro. willowae* sp. n.	2	–
N012	*Nicro. defodiens*	CAN, ON, Algonquin P.P. 1	45.902, -77.605	16.vi.08	*Macro. willowae* sp. n.	2	–
N013	*Nicro. defodiens*	CAN, ON, Algonquin P.P. 1	45.902, -77.605	16.vi.08	*Macro. willowae* sp. n.	5	–
N014	*Nicro. defodiens*	CAN, ON, Algonquin P.P. 1	45.902, -77.605	16.vi.08	*Macro. willowae* sp. n.	7	–
N015	*Nicro. defodiens*	CAN, ON, Algonquin P.P. 1	45.902, -77.605	16.vi.08	*Macro. willowae* sp. n.	5	–
N017	*Nicro. defodiens*	CAN, ON, Algonquin P.P. 1	45.902, -77.605	16.vi.08	*Macro. willowae* sp. n.	1	–
N018	*Nicro. defodiens*	CAN, ON, Algonquin P.P. 1	45.902, -77.605	16.vi.08	*Macro. willowae* sp. n.	2	–
N019	*Nicro. defodiens*	CAN, ON, Algonquin P.P. 1	45.902, -77.605	16.vi.08	*Macro. willowae* sp. n.	2	–
N020	*Nicro. orbicollis*	CAN, ON, Algonquin P.P. 1	45.902, -77.605	16.vi.08	*Macro. willowae* sp. n.	4	–
N021	*Nicro. defodiens*	CAN, ON, Algonquin P.P. 1	45.902, -77.605	16.vi.08	*Macro. willowae* sp. n.	1	–
N022	*Nicro. defodiens*	CAN, ON, Algonquin P.P. 1	45.902, -77.605	16.vi.08	*Macro. willowae* sp. n.	3	–
N023	*Nicro. defodiens*	CAN, ON, Algonquin P.P. 1	45.902, -77.605	16.vi.08	*Macro. willowae* sp. n.	2	–
N024	*Nicro. defodiens*	CAN, ON, Algonquin P.P. 1	45.902, -77.605	16.vi.08	*Macro. willowae* sp. n.	3	–
N026	*Nicro. orbicollis*	CAN, ON, Charleston Lake	44.500, -76.072	01.vii.08	*Macro. willowae* sp. n.	5	–
N028	*Nicro. defodiens*	CAN, ON, Algonquin P.P. 1	45.902, -77.605	30.vi.08	*Macro. willowae* sp. n.	3	–
N029	*Nicro. defodiens*	CAN, ON, Algonquin P.P. 1	45.902, -77.605	30.vi.08	*Macro. willowae* sp. n.	4	–
N031	*Nicro. defodiens*	CAN, ON, Algonquin P.P. 1	45.902, -77.605	30.vi.08	*Macro. willowae* sp. n.	2	–
N036	*Nicro. defodiens*	CAN, ON, Algonquin P.P. 1	45.902, -77.605	30.vi.08	*Macro. willowae* sp. n.	1	–
N037	*Nicro. defodiens*	CAN, ON, Algonquin P.P. 1	45.902, -77.605	30.vi.08	*Macro. willowae* sp. n.	3	–
N039	*Nicro. defodiens*	CAN, ON, Algonquin P.P. 1	45.902, -77.605	30.vi.08	*Macro. willowae* sp. n.	1	–
N040	*Nicro. defodiens*	CAN, ON, Algonquin P.P. 1	45.902, -77.605	30.vi.08	*Macro. willowae* sp. n.	8	–
N042	*Nicro. defodiens*	CAN, ON, Algonquin P.P. 1	45.902, -77.605	30.vi.08	*Macro. willowae* sp. n.	3	–
N043	*Nicro. defodiens*	CAN, ON, Algonquin P.P. 1	45.902, -77.605	30.vi.08	*Macro. willowae* sp. n.	3	–
N044	*Nicro. defodiens*	CAN, ON, Algonquin P.P. 1	45.902, -77.605	30.vi.08	*Macro. willowae* sp. n.	3	–
N046	*Nicro. defodiens*	CAN, ON, Algonquin P.P. 1	45.902, -77.605	30.vi.08	*Macro. willowae* sp. n.	1	–
N047	*Nicro. defodiens*	CAN, ON, Algonquin P.P. 1	45.902, -77.605	30.vi.08	*Macro. willowae* sp. n.	17	–
N048	*Nicro. orbicollis*	CAN, ON, Algonquin P.P. 1	45.902, -77.605	30.vi.08	*Macro. willowae* sp. n.	5	–
N051	*Nicro. orbicollis*	CAN, ON, Charleston Lake	44.500, -76.072	15.vii.08	*Macro. willowae* sp. n.	2	–
N052	*Nicro. orbicollis*	CAN, ON, Frontenac	44.447, -76.577	30.vii.08	Macro. willowae sp. n.	1	–
N055	*Nicro. orbicollis*	CAN, ON, Charleston Lake	44.500, -76.072	30.vii.08	*Macro. willowae* sp. n.	3	–
N057	*Nicro. defodiens*	CAN, ON, Algonquin P.P. 1	45.902, -77.605	29.vii.08	*Macro. willowae* sp. n.	1	–
N058	*Nicro. defodiens*	CAN, ON, Algonquin P.P. 1	45.902, -77.605	29.vii.08	*Macro. willowae* sp. n.	2	–
N060	*Nicro. defodiens*	CAN, ON, Algonquin P.P. 1	45.902, -77.605	29.vii.08	*Macro. willowae* sp. n.	2	–
N061	*Nicro. defodiens*	CAN, ON, Algonquin P.P. 1	45.902, -77.605	29.vii.08	*Macro. willowae* sp. n.	5	–
N062	*Nicro. defodiens*	CAN, ON, Algonquin P.P. 1	45.902, -77.605	29.vii.08	*Macro. willowae* sp. n.	3	–
N068	*Nicro. defodiens*	CAN, ON, Algonquin P.P. 1	45.902, -77.605	29.vii.08	*Macro. willowae* sp. n.	9	–
N069	*Nicro. orbicollis*	CAN, ON, Charleston Lake	44.500, -76.072	12.viii.08	*Macro. willowae* sp. n.	1	–
N075	*Nicro. orbicollis*	CAN, ON, Frontenac	44.447, -76.577	26.viii.08	*Macro. willowae* sp. n.	1	–
N081	*Nicro. carolinus*	USA, FL, Highlands Co, Lake Placid	27.181, -81.352	10.iii.2009	*Macro. kaiju* sp. n.	4	MF192750
N081	*Nicro. carolinus*	USA, FL, Highlands Co, Lake Placid	27.181, -81.352	10.iii.2009	*Macro. willowae* sp. n.	9	MF192743
N086	*Nicro. orbicollis*	CAN, ON, Windsor, Elgin St.	42.261, -83.057	18.vi.2009	*Macro. willowae* sp. n.	1	–
N088	*Nicro. orbicollis*	CAN, ON, Hwy 132, Dacre	45.369, -76.988	25.vi.2009	*Macro. willowae* sp. n.	10	–
N089	*Nicro. orbicollis*	CAN, ON, Carbine Rd.	45.33, -76.371	25.vi.2009	*Macro. willowae* sp. n.	9	–
N104	*Nicro. defodiens*	CAN, BC, Prince George, nr. UNBC	53.904, -122.783	12.vi.2009	*Macro. willowae* sp. n.	1	–
N110	*Nicro. defodiens*	CAN, BC, Prince George, nr. UNBC	53.904, -122.783	12.vi.2009	*Macro. willowae* sp. n.	1	–
N113	*Nicro. defodiens*	CAN, BC, Prince George, nr. UNBC	53.904, -122.783	12.vi.2009	*Macro. willowae* sp. n.	4	MF192748
N114	*Nicro. orbicollis*	CAN, PEI, Wellington, Route 2	46.452, -63.949	06.vii.2009	*Macro. willowae* sp. n.	8	–
N115	*Nicro. orbicollis*	CAN, PEI, Wellington, Route 2	46.452, -63.949	06.vii.2009	*Macro. willowae* sp. n.	7	–
N116	*Nicro. orbicollis*	CAN, PEI, Wellington, Route 2	46.452, -63.949	06.vii.2009	*Macro. praedafimetorum*	1	MF192754
N116	*Nicro. orbicollis*	CAN, PEI, Wellington, Route 2	46.452, -63.949	06.vii.2009	*Macro. willowae* sp. n.	9	MF192747
N136	*Nicro. defodiens*	CAN, BC, Prince George, nr. UNBC	53.904, -122.783	12.vi.2009	*Macro. willowae* sp. n.	1	MF192749
N139	*Nicro. orbicollis*	CAN, ON, Carbine Rd.	45.33, -76.371	10.vii.2009	*Macro. willowae* sp. n.	12	–
N143	*Nicro. orbicollis*	CAN, ON, Hamilton, Site 4-7		07.vii.2009	*Macro. willowae* sp. n.	100	MF192744
N145	*Nicro. orbicollis*	CAN, ON, Hamilton, Site 4-7		07.vii.2009	*Macro. willowae* sp. n.	8	–
N147	*Nicro. orbicollis*	CAN, ON, Hamilton, Site 4-7		07.vii.2009	*Macro. willowae* sp. n.	10	–
N153	*Nicro. orbicollis*	CAN, NS, Dartmouth, Wright’s Cove Rd.	44.694, -63.611	09.vii.2009	*Macro. willowae* sp. n.	4	–
N160	*Nicro. orbicollis*	CAN, ON, Waterloo	43.54, -80.211	07.vii.2009	*Macro. willowae* sp. n.	2	–
N161	*Nicro. orbicollis*	CAN, ON, Waterloo	43.54, -80.211	07.vii.2009	*Macro. willowae* sp. n.	8	–
N165	*Nicro. orbicollis*	CAN, ON, Carbine Rd.	45.33, -76.371	23.vii.2009	*Macro. willowae* sp. n.	17	–
N166	*Nicro. orbicollis*	CAN, ON, Carbine Rd.	45.33, -76.371	23.vii.2009	*Macro. willowae* sp. n.	8	–
N167	*Nicro. orbicollis*	CAN, NS, Glenmont, Black Hole Rd.	45.111, -64.296	17.vii.2009	*Macro. willowae* sp. n.	9	–
N168	*Nicro. orbicollis*	CAN, NS, Cold Brook, Hwy 101	45.079, -64.592	13.vii.2009	*Macro. willowae* sp. n.	10	MF192745
N169	*Nicro. orbicollis*	CAN, NS, Chipman	46.174, -65.899	08.vii.2009	*Macro. willowae* sp. n.	7	–
N174	*Nicro. orbicollis*	CAN, NS, Hantsport	45.099, -64.184	21.vii.2009	*Macro. willowae* sp. n.	6	–
N178	*Nicro. orbicollis*	CAN, NS, East River off Hwy 329	44.583, -64.164	10.viii.2009	*Macro. willowae* sp. n.	4	–
N180	*Nicro. defodiens*	CAN, NS, Debert, Industrial Park	45.428, -63.429	05.viii.2009	*Macro. willowae* sp. n.	3	–
N181	*Nicro. orbicollis*	CAN, ON, Carbine Rd.	45.33, -76.371	06.viii.2009	*Macro. willowae* sp. n.	8	MF192746
N185	*Nicro. vespillo*	GER, Mooswald Forest, nr. Freiburg	48.0, 7.85	vi.2009	*Macro. nataliae*	4	–
N186	*Nicro. vespillo*	GER, Mooswald Forest, nr. Freiburg	48.0, 7.85	vi.2009	*Macro. nataliae*	7	MF192752
N187	*Nicro. vespillo*	GER, Mooswald Forest, nr. Freiburg	48.0, 7.85	vi.2009	*Macro. nataliae*	1	–
N188	*Nicro. vespillo*	GER, Mooswald Forest, nr. Freiburg	48.0, 7.85	vi.2009	*Macro. nataliae*	5	MF192753
N191	*Nicro. orbicollis*	USA, CT, Bethany	41.462, -72.961	16.vii.2009	*Macro. willowae* sp. n.	1	–
N192	*Nicro. orbicollis*	USA, CT, Bethany	41.462, -72.961	14.viii.2009	*Macro. willowae* sp. n.	5	–
N216	*Nicro. orbicollis*	USA, NH, Durham	43.134, -70.926	07.vi.2009	*Macro. willowae* sp. n.	215	–
N218	*Nicro. orbicollis*	USA, NH, Durham	43.134, -70.926	07.vi.2009	*Macro. willowae* sp. n.	135	–
N222	*Nicro. defodiens*	USA, NH, Durham	43.134, -70.926	07.vi.2009	*Macro. willowae* sp. n.	30	–
N226	*Nicro. orbicollis*	USA, NH, Durham	43.134, -70.926	07.vi.2009	*Macro. willowae* sp. n.	30	–
N228	*Nicro. orbicollis*	USA, NH, Durham	43.134, -70.926	07.vi.2009	*Macro. willowae* sp. n.	97	–
N234	*Nicro. marginatus*	CAN, AB, Onefour	49.121, -110.47	17.vi.2003	*Macro. pratum* sp. n.	10	–
N235	*Nicro. guttula*	CAN, AB, Onefour	49.121, -110.47	17.vi.2003	*Macro. pratum* sp. n.	7	–
N235x	*Nicro. marginatus*	CAN, AB, Onefour	49.121, -110.47	04.vii.2002	*Macro. pratum* sp. n.	3	–
N236	*Nicro. obscurus*	CAN, AB, Onefour	49.121, -110.47	04.vii.2002	*Macro. pratum* sp. n.	5	–
N237	*Nicro. marginatus*	CAN, AB, Onefour	49.121, -110.47	17.vi.2003	*Macro. pratum* sp. n.	13	–
N238	*Nicro. obscurus*	CAN, AB, Onefour	49.121, -110.47	17.vi.2003	*Macro. pratum* sp. n.	5	–
N239	*Nicro. obscurus*	CAN, AB, Onefour	49.121, -110.47	17.vi.2003	*Macro. pratum* sp. n.	11	–
N240	*Nicro. marginatus*	CAN, AB, Onefour	49.121, -110.47	17.vi.2003	*Macro. pratum* sp. n.	13	–
N242	*Nicro. hybridus*	CAN, AB, Onefour	49.121, -110.47	18.vii.2002	*Macro. pratum* sp. n.	7	MF192751
N243	*Nicro. guttula*	CAN, AB, Onefour	49.121, -110.47	17.vi.2003	*Macro. pratum* sp. n.	7	–
N244	*Nicro. guttula*	CAN, AB, Onefour	49.121, -110.47	17.vi.2003	*Macro. pratum* sp. n.	7	–
N246	*Nicro. hybridus*	CAN, AB, Onefour	49.121, -110.47	18.vii.2002	*Macro. pratum* sp. n.	10	–
N274	*Nicro. marginatus*	CAN, AB, Onefour	49.121, -110.47	17.vi.2003	*Macro. glaber*	8	–
N275	*Nicro. obscurus*	CAN, AB, Onefour	49.121, -110.47	17.vi.2003	*Macro. glaber*	1	–
N295	*Nicro. carolinus*	USA, NE, Kearney Co.		05.vi.2009	*Macro. kaiju* sp. n.	1	–
N295	*Nicro. carolinus*	USA, NE, Kearney Co.		05.vi.2009	*Macro.* sp.	4	–
N298	*Nicro. carolinus*	USA, NE, Kearney Co.		05.vi.2009	*Macro. kaiju* sp. n.	2	–
N298	*Nicro. carolinus*	USA, NE, Kearney Co.		05.vi.2009	*Macro.* sp.	3	–
N303	*Nicro. pustulatus*	USA, NE, Kearney Co.		13.vii.2009	*Macro. pratum* sp. n.	3	–
N308	*Nicro. marginatus*	CAN, AB, Onefour	49.121, -110.47	17.vi.2003	*Macro. pratum* sp. n.	86	–
N329	*Nicro. orbicollis*	USA, NH		2009	*Macro. willowae* sp. n.	41	–
N330	*Nicro. orbicollis*	USA, NH		2009	*Macro. willowae* sp. n.	91	–
N331	*Nicro. orbicollis*	USA, CT, Bethany	41.462, -72.961	2009	*Macro. willowae* sp. n.	13	–
N332	*Nicro. carolinus*	USA, FL, Highlands Co, Lake Placid	27.181, -81.352	10.iii.2009	*Macro. kaiju* sp. n.	11	–
N333	*Nicro. carolinus*	USA, FL, Highlands Co, Lake Placid	27.181, -81.352	10.iii.2009	*Macro. kaiju* sp. n.	74	–
N334	*Nicro. carolinus*	USA, FL, Highlands Co, Lake Placid	27.181, -81.352	10.iii.2009	*Macro. willowae* sp. n.	1	–
N334	*Nicro. carolinus*	USA, FL, Highlands Co, Lake Placid	27.181, -81.352	10.iii.2009	*Macro. kaiju* sp. n.	26	–
N334	*Nicro. carolinus*	USA, FL, Highlands Co, Lake Placid	27.181, -81.352	10.iii.2009	*Macro.* sp.	1	–
N335	*Nicro. carolinus*	USA, FL, Highlands Co, Lake Placid	27.181, -81.352	10.iii.2009	*Macro. kaiju* sp. n.	45	–
N336	*Nicro. marginatus*	USA, NE, Kearney Co.		vi.2009	*Macro. pratum* sp. n.	9	–
N338	*Nicro. defodiens*	USA, NH, Durham	43.134, -70.926	07.vi.2009	*Macro. willowae* sp. n.	8	–
N339	*Nicro. defodiens*	USA, NH, Durham	43.134, -70.926	07.vi.2009	*Macro. willowae* sp. n.	163	–
N346	*Nicro. orbicollis*	CAN, ON, Hwy 132, Dacre	45.369, -76.988	06.viii.2009	*Macro. willowae* sp. n.	6	–
N347	*Nicro. orbicollis*	CAN, ON, Carbine Rd.	45.33, -76.371	06.viii.2009	*Macro. willowae* sp. n.	16	–

Pairwise distances were calculated using neighbour-joining (NJ) analyses with the Kimura-2-parameter (K2P) model in PAUP* v4.0b10 (Swofford 2003). Phylogenetic reconstructions of the COI dataset was performed using Bayesian inference (BI) in MrBayes v3.2.6 (Huelsenbeck and Ronquist 2001; Ronquist and Huelsenbeck 2003). Each specimen in the phylogeny is labeled with the mite species and the beetle number, followed by the host species and abbreviated state, province or country (Fig. 14).

MrModeltest v2.3 (Nylander 2004) was used to determine the best-fit model of molecular evolution for each molecular marker, which was determined to be GTR+I+G. Bayesian analysis was performed in MrBayes using the Markov Chain Monte Carlo (MCMC) method, two independent runs, with nucmodel = 4by4, N_st_ = 6, rates = invgamma, samplefreq = 1000, four chains = one cold and three heated. The COI dataset ran for 10 million generations, producing 19502 trees after a burn-in of 250 trees. The remaining trees in Mesquite, excluding the burn-in, were used to generate a majority-rule consensus tree displaying the posterior probability supports for each node. Bayesian analyses were performed using the on-line Computational Biology Service Unit at Cornell University, and at the Cyberinfrastructure for Phylogenetic Research (CIPRES) portal (Miller et al. 2010).

## Results and discussion

### Family Macrochelidae Vitzthum, 1930

#### Subfamily 

##### Genus Macrocheles

Taxon classificationAnimaliaMesostigmataMacrochelidae

Latreille, 1829

###### Type species.

*Acarus marginatus* Hermann, 1804 (= *Acarus muscae domesticae* Scopoli, 1772), by original designation.

##### Macrocheles willowae sp. n.

Taxon classificationAnimaliaMesostigmataMacrochelidae

http://zoobank.org/1085F03B-0DC9-4762-91E4-6FA1770E6965

**Figure 1.**
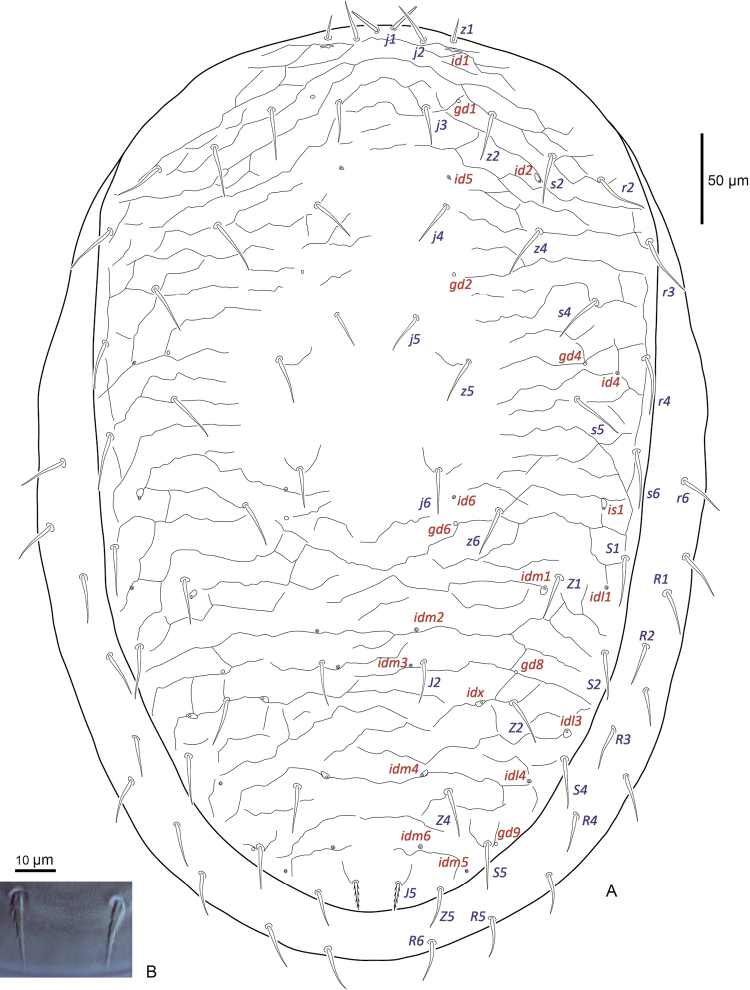
Female *Macrocheles willowae* sp. n. **A** dorsal idiosoma **B** seta *J5*.

**Figure 2.**
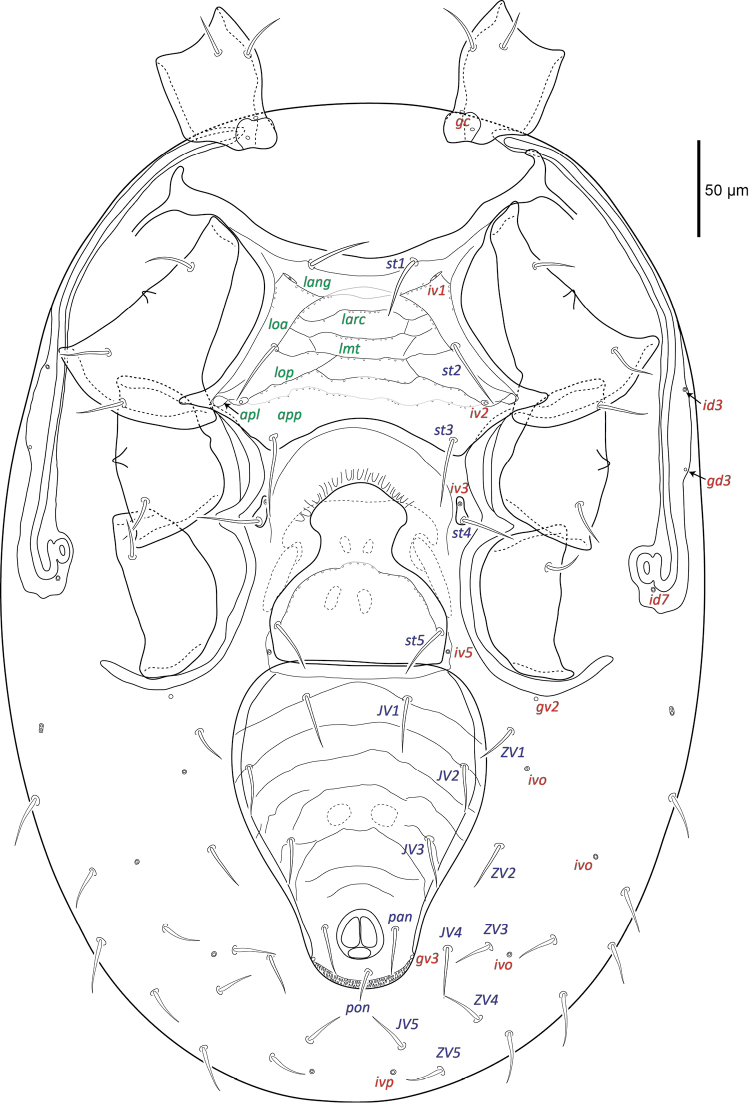
Female *Macrocheles willowae* sp. n. ventral idiosoma including coxae.

**Figure 3.**
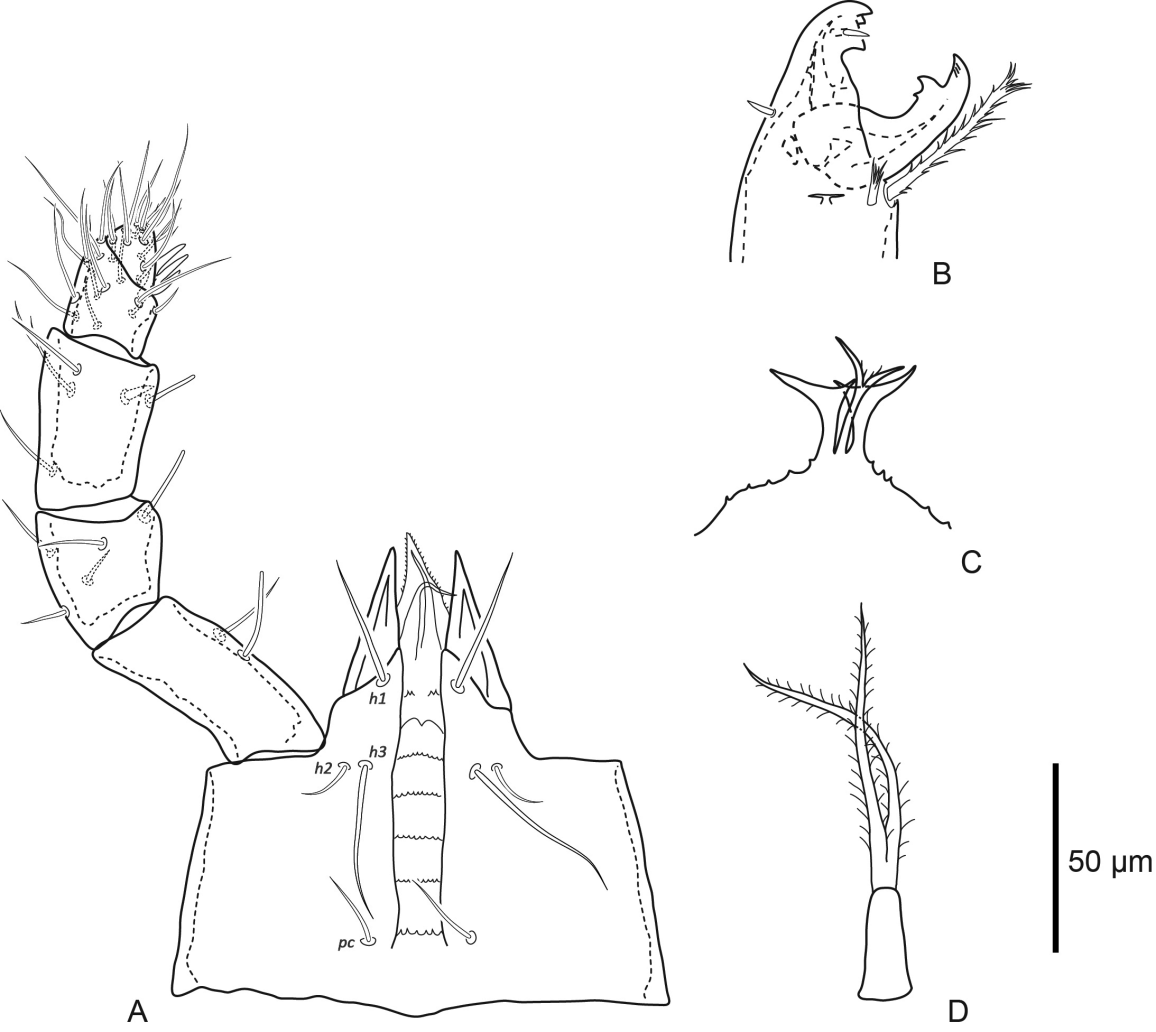
Female *Macrocheles willowae* sp. n. **A** subcapitulum and palp, ventral aspect **B** chelicera, antiaxial aspect **C** epistome **D** tritosternum.

**Figure 4.**
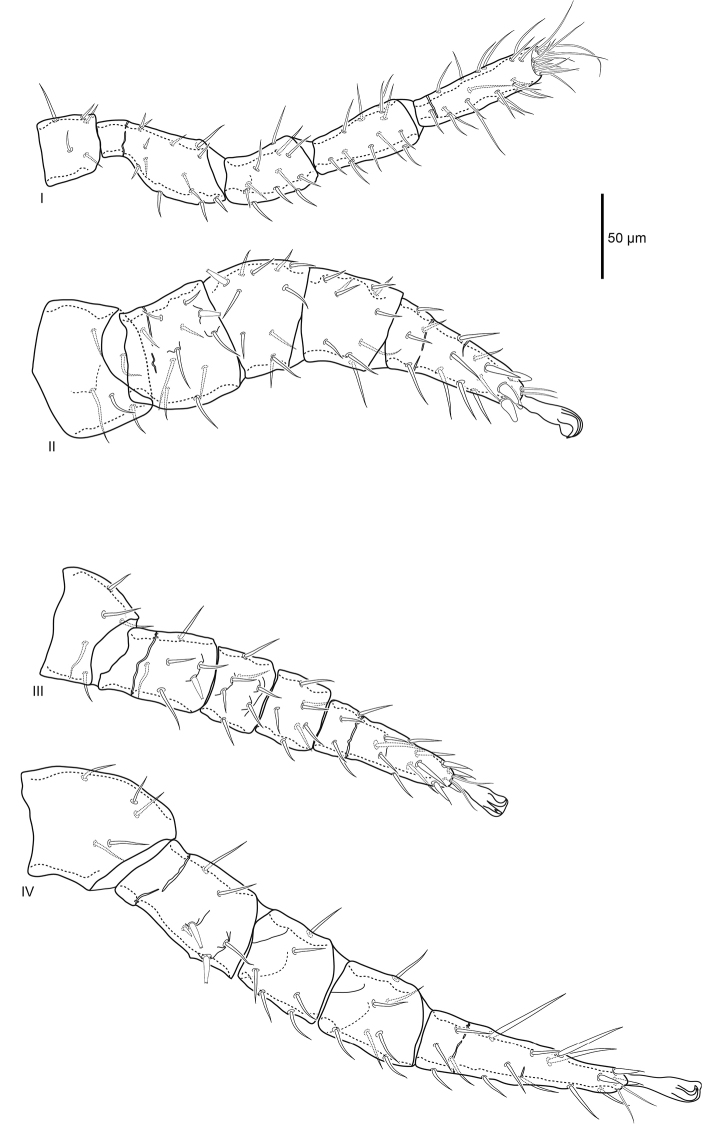
Female *Macrocheles willowae* sp. n. legs I–IV, coxae omitted; leg I anterolateral, II posterolateral, III dorsal, IV anterolateral.

Figs 1 , 2 , 3 , 4 , 13A

###### Material examined.

***Type material*.** Holotype: female (CNC829414) on *Nicrophorus orbicollis* (N088, female) collected near Dacre, Ontario, Canada (45.369, -76.988), 25.vi.2009, coll: W. Knee.

Paratypes (26): Nine females (CNC829415–829423) with the same collection information as the holotype; 15 females (CNC829424–829438) on *N. defodiens* (N222, female), Durham, New Hampshire, USA (43.134, -70.926), 07.vi.2009, coll: W. Knee & M. Scott; female (CNC829439) on *N. defodiens* (N136, female), Prince George, near University of Northern British Columbia campus, British Columbia, Canada (53.904, -122.783), 12.vi.2009, coll: W. Knee & R. Dawson; female (CNC829440) on *N. orbicollis* (N143, male), Hamilton, Ontario, Canada, 7.vii.2009, coll: W. Knee.

***Other material*.** 1241 mites examined from British Columbia, Nova Scotia, Ontario, Prince Edward Island, Connecticut, Florida, and New Hampshire on *N. carolinus, N. defodiens*, and *N. orbicollis* (Table 1).

###### Diagnosis female.

As for *Macrocheles* (see Hyatt and Emberson 1988). All dorsal and ventral setae smooth and spinose, except *J5* barbed and slightly shorter than *Z5*. Seta *j1* simple with rounded tip, *j1* slightly longer than *z1*. Dorsal hexagonal setae (*j5, z5, j6*) nearly as long as marginal and submarginal setae (*R* and *UR*). Dorsal shield with moderate reticulations throughout, except smooth in dorsal hexagonal area and between *j4* setae, without well-defined procurved line, sigillary rami absent. Sternal shield more than twice as wide as long, punctures small, posterior margin concave. Well defined *linea media transversa* (l.m.t.) and *linea oblique anteriores* (l.o.a.), l.o.a. contacts l.m.t. *Linea arcuata* (l.arc.) well defined and contacts l.o.a. *Linea angulata* (l.ang.) and *linea oblique posteriore* (l.o.p.) well defined laterally but faint medially. *Area punctata laterale* (a.p.l.) well defined, but *area punctata posteriore* (a.p.p.) not well defined. Ventrianal shield longer than wide (ratio 1.3). Arthrodial brush as long as movable digit. Genu IV with six setae. Femur IV setae *ad2*, *pd1* prominent spikes with flattened forked tip.

###### Description female.

***Dorsal idiosoma*** (Fig. 1). Dorsal shield 548 (526–572) long and 357 (344–372) (n=8) wide (level with *r3*), with 28 pairs of setae, all setae simple and spinose except *J5* is barbed. Seta *J5* 16 (15–18) shorter than *Z5* 24 (22–27). Seta *j1* simple with rounded tip, *j1* 18 (16–20) slightly longer than *z1* 16 (12–19). Marginal and submarginal setae simple, slightly longer 24 (23–25) than dorsal hexagonal setae 20 (15–22). Dorsal shield with moderate reticulations throughout, except smooth in dorsal hexagonal area and between *j4* setae, shield without well-defined procurved line, sigillary rami absent, and posterolateral margins narrowed slightly. Shield with 22 pairs of pore-like structures, of which six are secretory glands and 16 are non-secretory poroids.

***Ventral idiosoma*** (Figs 2, 13A). Sternal shield more than twice as wide as long, medial length 91 (88–96), maximum width 213 (192–221) level with a.p.l., and minimum width 117 (115–124) posterior of *st1*. Sternal shield punctures small, posterior margin concave. Setae *st1–3* 38 (33–43) simple and spinose, and two pairs of lyrifissures (*iv1*, *iv2*) on sternal shield. Pear-shaped metasternal shields well separated from sternal shield margin bearing lyrifissure *iv3* anteriorly and spinose seta *st4* 33 (30–35) posteriorly. Well defined l.m.t. and l.o.a., l.o.a. contacts l.m.t. Well defined l.arc. contacts l.o.a., l.ang. and l.o.p. well defined laterally but faint medially. Well defined a.p.l., a.p.p. not well defined. Genital shield length 149 (141–161), width 113 (104–120) level with *st5*. Genital shield truncate posteriorly and hyaline margin rounded anteriorly, spinose seta *st5* 32 (30–34) on shield, pair of lyrifissures *iv5* off shield near posterior margin. Transverse line on genital shield well defined laterally and faint medially, small punctures along transverse line. Peritrematal shield narrow, fused to dorsal shield near *r3*, peritreme extends beyond posterior margin of coxa I, two poroids (*id3*, *id7*) and one gland (*gd3*) on the shield. Ventrianal shield longer than wide (ratio 1.3); length 198 (187–204), width 153 (144–168) level with *JV2*. Ventrianal shield bearing several faint transverse lines, three pairs of simple spinose preanal setae *JV1–JV3* 26 (21–30), spinose paranal (*pan*) 27 (25–30) and postanal (*pon*) 18 (16–20) setae, narrow cribrum and a pair of glands (*gv3*) on shield margin posterior of the anal opening. Ventral opisthosomal setae in soft integument simple and spinose, *ZV1* 21 (17–29), *ZV2* 26 (22–29) as long or nearly as long as *Jv* setae. Two pairs of glands (*gv2* and unknown paired-pore) and four pairs of poroids (*ivo*, *ivp*) in opisthosomal soft integument.

***Gnathosoma*** (Fig. 3). Basis capitulum medial length excluding internal malae 114 (106–122), width 150 (143–157) posterior to *pc.* Subcapitular setae simple: *h1* 38 (28–48), *h2* 16 (13–19), *h3* 53 (44–67), and *pc* 19 (16–21). Palp chaetotaxy normal for genus (2–5–6–14–15), palp apotele three-tined, *al* setae on trochanter, femur and genu slightly spatulate. Corniculi pointed, length along lateral margin 42 (37–51), internal malae slender and smooth. Epistome tripartite with bifid central element bearing small fringe medially, lateral elements broad and flag-like distally, epistomatic margin finely serrate. Subcapitulum with seven rows, six of which have deutosternal denticles; the anterior most row with few (four) denticles laterally, and the second anterior most row with paired ridges without any denticles. Chelicerae robust, length of second cheliceral segment including fixed digit 140 (134–146), and movable digit 54 (52–58). Fixed digit bidentate with one large and one small tooth, moveable digit with bidentate tooth. Pilus dentilis and dorsal seta on fixed digit simple spikes, fixed digit with lyrifissure on each paraxial and antiaxial faces. Movable digit with narrow fringed arthrodial corona, and plumose arthrodial brush (50) almost as long as movable digit.

***Legs*** (Fig. 4). Excluding ambulacra, lengths of leg I 420 (409–430), leg II 383 (357–402), leg III 338 (315–349), and leg IV 482 (469–489). As in all *Macrocheles*, ambulacra only present on legs II–IV, claws II–IV well developed. Pair of glands (*gc*) on coxa I. Setation of legs I–IV normal for Macrochelidae: coxae 2–2–2–1; trochanters 5–5–5–5; femora I (2–3/1,2/3–2) (as *al–ad/av, pd/pv–pl*), II (2–3/1,2/2–1), III (1–2/0,1/1–1), IV (1–2/1,1/0–1); genua I, II (2–3/1,2/1–2), III (1–2/1,2/0–1), IV (1–2/1,2/0–0); tibiae I (2–3/2,2/1–2), II (2–2/1,2/1–2), III, IV (1–1/1,2/1–1); tarsus I 20 setae plus numerous tapered setae distally, tarsi II–IV 18. Most leg setae simple, setiform, femur II *ad1*, III *pd1*, IV *ad2*, *pd1*, and genu II *ad3* prominent spike setae with flattened forked tip with two to four tines that can appear as a single tapered point viewed laterally. Tarsus II with four large distal spike setae with thickened conical base and rounded tip. Tarsi III, IV with four and three, respectively, distal setae with wide base and flexible filamentous tip. Genu and tibia IV with paired slight ridge anterolateral and posterolateral.

###### Male and immatures.

Unknown.

###### Etymology.

This species is named after my daughter Willow Knee. May it inspire her to notice the little creatures as well as the big.

###### Remarks.

*Macrocheles willowae* sp. n. is most similar to *M. merdarius* (Berlese), *M. nemerdarius* Krantz and Whitaker, and *M. pratum* sp. n. *Macrocheles merdarius* is frequently found in litter, manure and compost worldwide, feeding on nematodes and eggs of insects (Krantz and Whitaker 1988). *Macrocheles merdarius* has been reported from small mammals, and female mites are often phoretic associates of dung beetles (Filipponi and Pegazzano 1963, Krantz and Whitaker 1988). Female *M. willowae* sp. n. differs from that of *M. merdarius* in the shape of the ventrianal shield, shape of the sternal shield, and length of the arthrodial brush. The anterior margin of the ventrianal shield is more truncated, and the widest part of the shield near *JV2* is more angular for *M. merdarius* than it is for *M. willowae* sp. n. The posterior margin of the sternal shield is more concave for *M. willowae* sp. n. The arthrodial brush is almost as long as the movable digit for *M. willowae* sp. n., and for *M. merdarius* the brush is approximately half as long as the movable digit. Comparisons were made using the species description for *M. merdarius* and slide mounted material deposited in the CNC.

*Macrocheles nemerdarius* was described from the nest of a mouse, *Peromyscus* in Maryland and the nest of the eastern woodrat *Neotoma floridana* in Florida, USA, and this species is also phoretic on coprophilous beetles (Krantz and Whitaker 1988). Female *M. willowae* sp. n. differs from that of *M. nemerdarius* in having marginal or submarginal setae slightly longer than dorsal hexagonal setae, posterior margin of sternal shield more concave, *pon* seta smooth not weakly pilose, *j1* only slightly longer than *z1* not 1.5 times as long, and *J5* slightly shorter than *Z5* not half as long as *Z5*. Comparisons were made using the species description for *M. nemerdarius* and examination of the holotype specimen loaned from the National Museum of Natural History, Smithsonian Institution.

Female *M. willowae* sp. n. differs from that of *M. pratum* sp. n. in having marginal and submarginal setae slightly longer than dorsal hexagonal setae. Genu and tibia IV with slight ridge on anterolateral and posterolateral surfaces in *M. willowae* sp. n., while *M. pratum* sp. n. only has a ridge on the posterolateral surface. Seta *J5* is slightly shorter than *Z5* and more spinose in *M. willowae* sp. n., *J5* is less than half as long as *Z5* in *M. pratum* sp. n. Punctures on the sternal shield are smaller and less prominent in *M. willowae* sp. n. than in *M. pratum* sp. n. The ventrianal shield is longer than wide for both species, but the shield is slightly narrower in *M. pratum* sp. n., ratio of 1.4 compared to 1.3 for *M. willowae* sp. n.

In Krantz and Whitaker (1988), Dr. W. Yoder provided a short diagnosis and partial illustrations of the female and male of an undescribed and unnamed *Macrocheles* species collected from *Nicrophorus* beetles and three mammal species (*Tamiasciurus hudsonius*, American red squirrel in Michigan; *Tamias striatus*, eastern chipmunk in Maryland; and *Zapus hudsonius*, meadow jumping mouse in Prince Edward Island). This undescribed species was a common associate of *Nicrophorus* beetles, but it was also found frequently enough on live rodents to suggest an association with small mammals (Krantz and Whitaker 1988). Dr. W. Yoder reportedly intended to formally describe and illustrate this new species of *Macrocheles*; however, to date this species has not been described. *Macrocheles willowae* sp. n. is likely the same species that Dr. W. Yoder was intending to describe. Over several years, repeated attempts were made to contact Dr. W. Yoder about the status of the description, but contact was unsuccessful.

##### Macrocheles pratum sp. n.

Taxon classificationAnimaliaMesostigmataMacrochelidae

http://zoobank.org/036A8E8D-9669-4524-89B4-4395BC71385A

**Figure 5.**
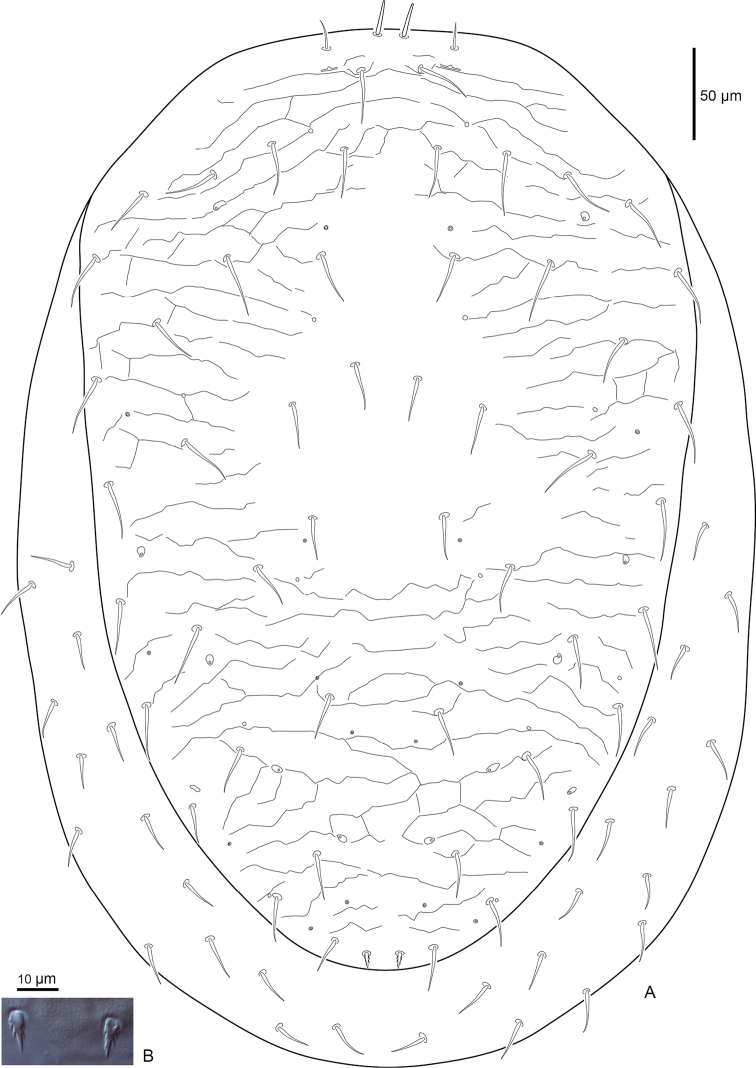
Female *Macrocheles pratum* sp. n. **A** dorsal idiosoma **B** seta *J5*.

**Figure 6.**
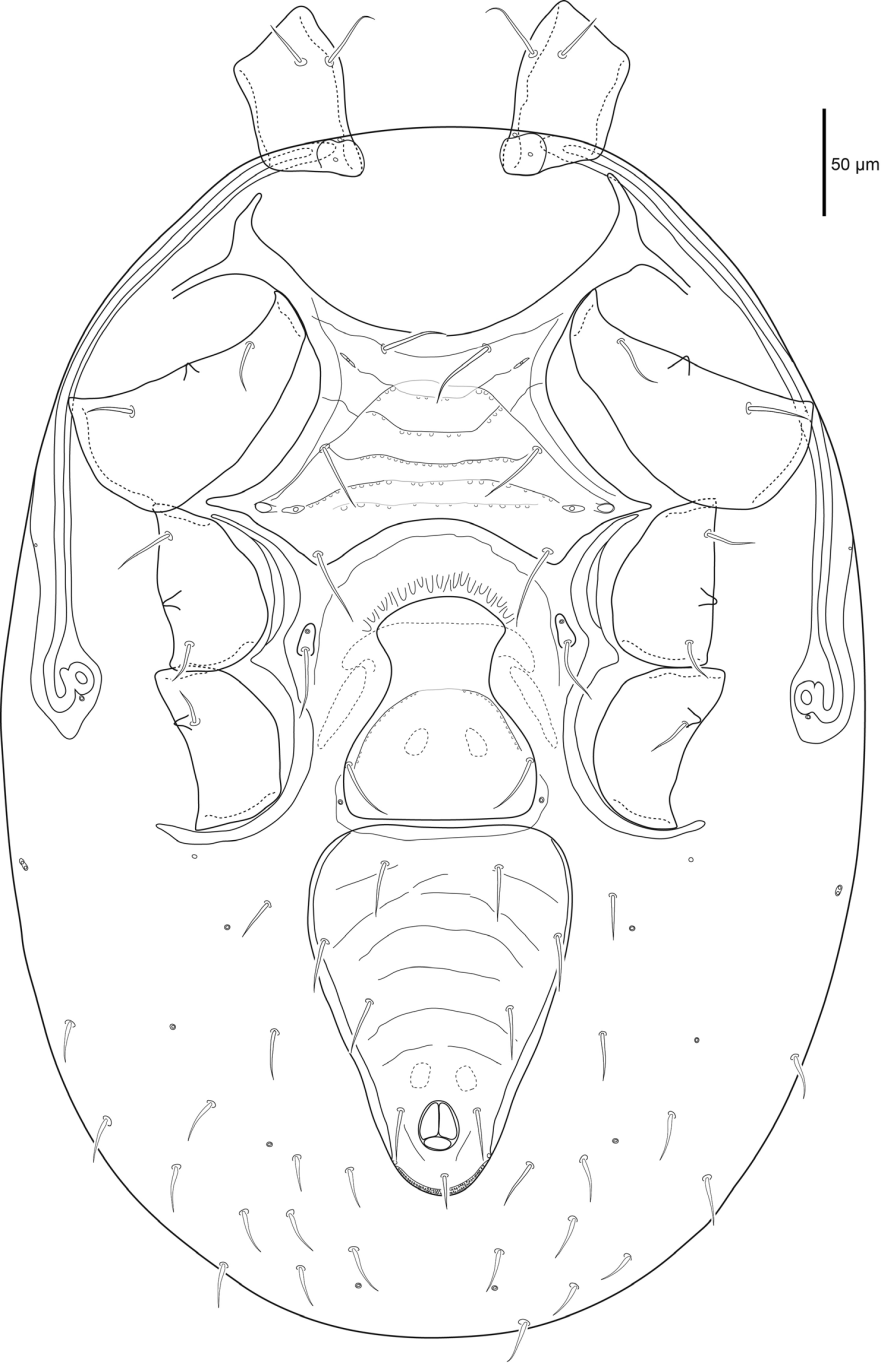
Female *Macrocheles pratum* sp. n. ventral idiosoma including coxae.

**Figure 7.**
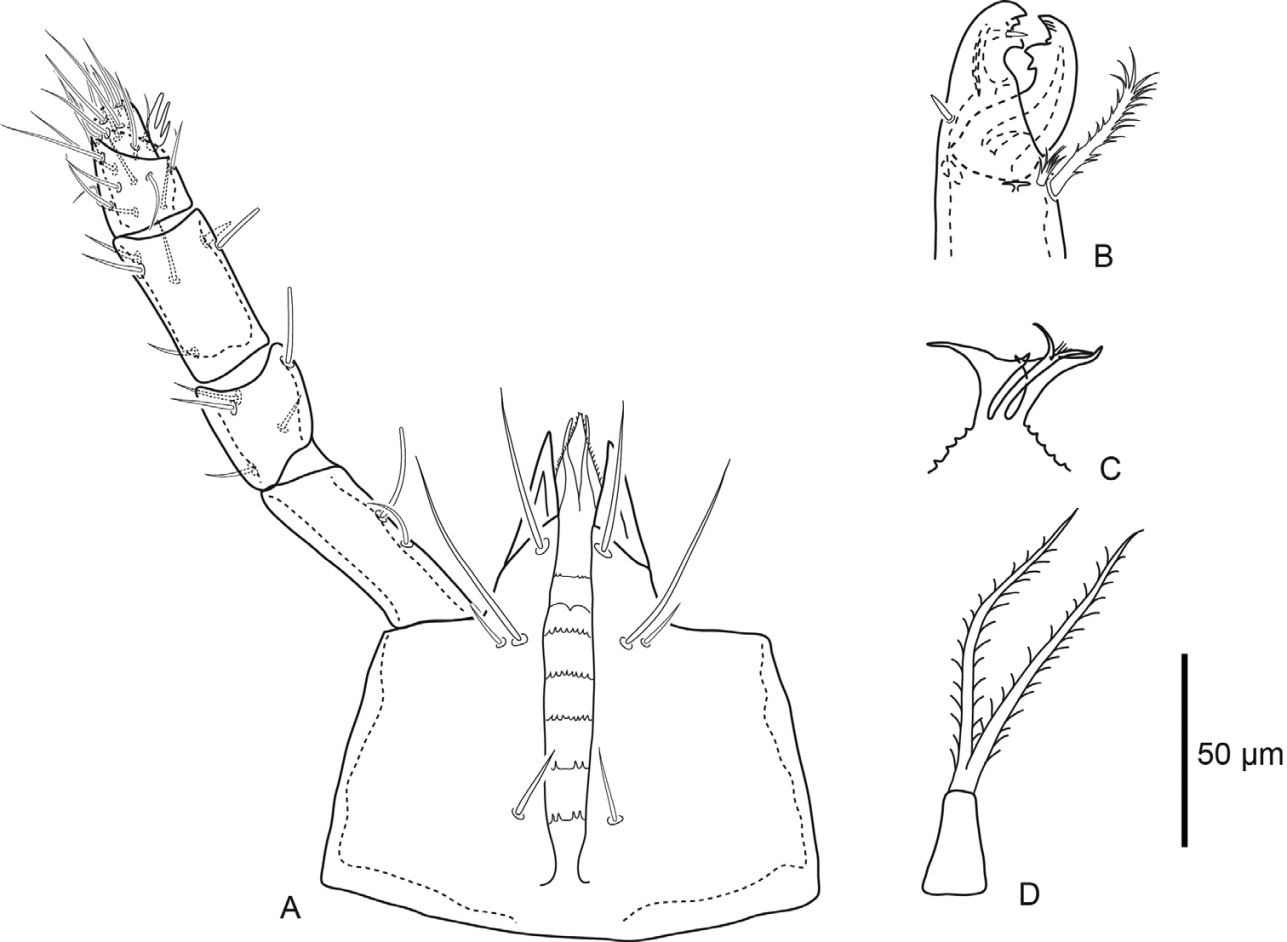
Female *Macrocheles pratum* sp. n. **A** subcapitulum and palp, ventral aspect **B** chelicera, antiaxial aspect **C** epistome **D** tritosternum.

**Figure 8.**
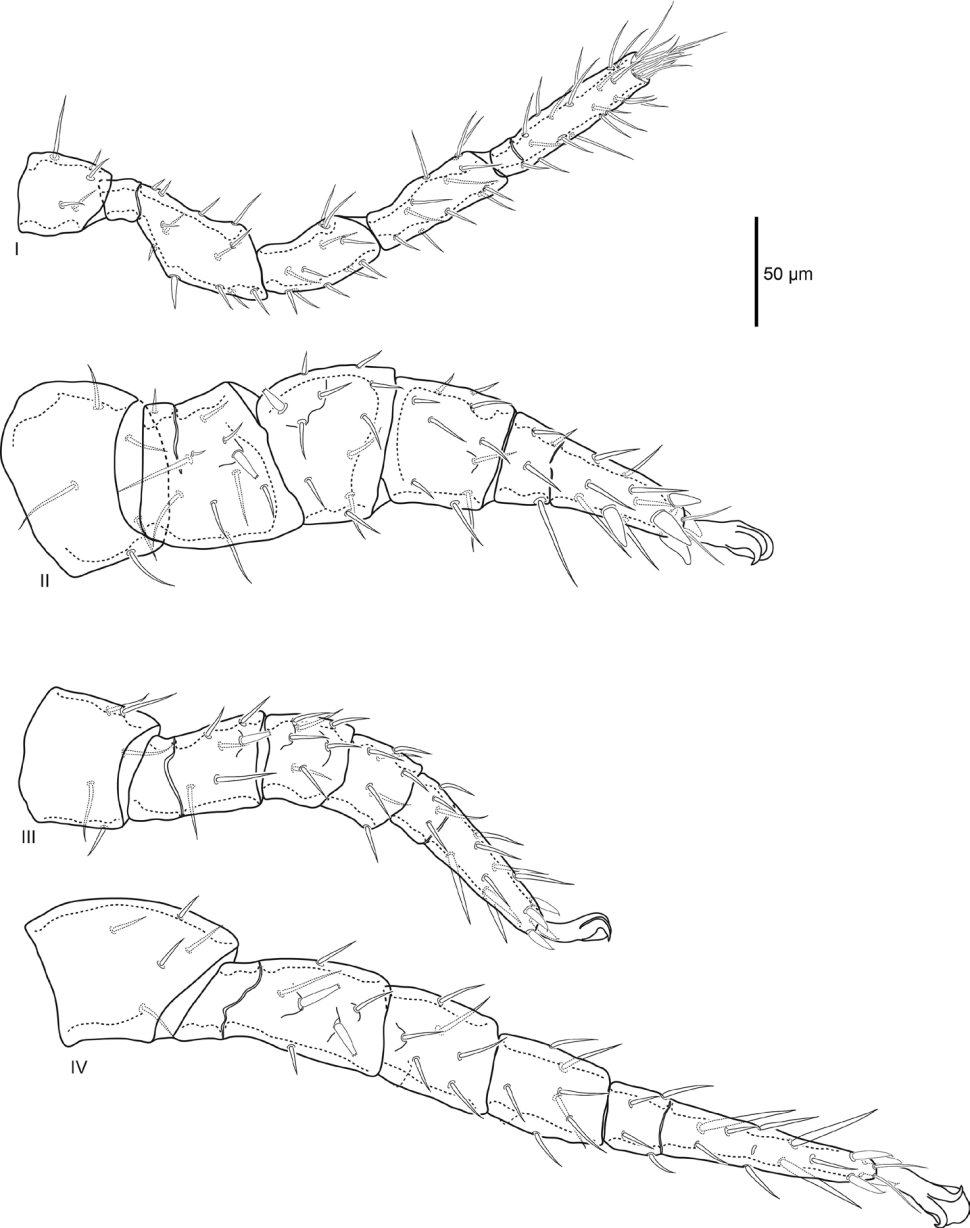
Female *Macrocheles pratum* sp. n. legs I–IV, coxae omitted; leg I anterolateral, II posterolateral, III posterolateral, IV femur dorsal, genu, tibia, tarsus anterolateral.

Figs 5 , 6 , 7 , 8 , 13B

###### Material examined.

***Type material*.** Holotype: female (CNC829441) on *Nicrophorus marginatus* (N336, female) collected in Kearney Co., Nebraska, USA, vi.2009, coll: W. Knee & W. Hoback.

Paratypes (11): eight females (CNC829442–829449) with the same collection information as the holotype; two females (CNC829450, 829451) on *N. hybridus* (N242, female), Onefour, Alberta, Canada, 18.vii.2002, coll: W. Knee & D. Johnson; female (CNC829452) on *N. guttula* (N235, female), Onefour, Alberta, 17.vi.2003, coll: W. Knee & D. Johnson.

***Other material*.** 184 mites examined from Alberta and Nebraska on *Nicrophorus guttula*, *N. hybridus*, *N. marginatus*, *N. obscurus*, and *N. pustulatus* (Table 1).

###### Diagnosis female.

All dorsal and ventral setae smooth and spinose, except *J5* barbed and much shorter than *Z5*. Seta *j1* simple with rounded tip, *j1* slightly longer than *z1*. Dorsal hexagonal setae slightly longer than marginal and submarginal setae. Dorsal shield with moderate reticulations throughout, except smooth in dorsal hexagonal area and between *j4* setae, without well-defined procurved line, sigillary rami absent. Sternal shield more than twice as wide as long, punctures moderate size, posterior margin concave. Well defined l.m.t. and l.o.a.; l.o.a. contacts l.m.t. Well defined l.arc. contacts l.o.a., l.ang. and l.o.p. well defined laterally but faint medially. Well defined a.p.l., but a.p.p. not well defined. Ventrianal shield longer than wide (ratio 1.4). Arthrodial brush as long as movable digit. Genu IV with six setae. Femur IV setae *ad2*, *pd1* prominent spikes with flattened forked tip.

###### Description female.

***Dorsal idiosoma*** (Fig. 5). Dorsal shield 520 (469–547) long and 358 (323–379) (n=8) wide (level with *r3*), with 28 pairs of setae, all setae simple and spinose except *J5* barbed. Seta *J5* 9 (8–10) half as long as *Z5* 22 (18–23). Seta *j1* simple with rounded tip, *j1* 20 (19–21) longer than *z1* 16 (13–18). Marginal and submarginal setae simple, shorter (19) than dorsal hexagonal setae 25 (24–28). Dorsal shield with moderate reticulations throughout, except smooth in dorsal hexagonal area and between *j4* setae, shield without well-defined procurved line, sigillary rami absent, and posterolateral margins narrowed slightly. Shield with 22 pairs of pore-like structures, of which six are secretory glands and 16 are non-secretory poroids.

***Ventral idiosoma*** (Figs 6, 13B). Sternal shield more than twice as wide as long, medial length 92 (86–99), maximum width 217 (196–224) level with a.p.l., and minimum width 114 (110–119) posterior of *st1*. Sternal shield punctures moderate size, posterior margin concave. Setae *st1–3* 40 (35–45) simple and spinose, and two pairs of lyrifissures (*iv1*, *iv2*) on sternal shield. Pear-shaped metasternal shields well separated from sternal shield margin bearing lyrifissure *iv3* anteriorly and spinose seta *st4* 35 (33–39) posteriorly. Well defined l.m.t. and l.o.a., l.o.a. contacts l.m.t. Well defined l.arc. contacts l.o.a., l.ang. and l.o.p. well defined laterally but faint medially. Well defined a.p.l., a.p.p. not well defined. Genital shield length 151 (138–158), width 112 (99–122) level with *st5*. Genital shield truncate posteriorly and hyaline margin rounded anteriorly, spinose seta *st5* 32 (30–34) on shield, pair of lyrifissures *iv5* off shield near posterior margin. Transverse line on genital shield well defined laterally and faint medially, small punctures along transverse line. Peritrematal shield narrow, fused to dorsal shield near *r3*, peritreme extends beyond posterior margin of coxa I, two poroids (*id3*, *id7*) and one gland (*gd3*) on the shield. Ventrianal shield longer than wide (ratio 1.4); length 186 (168–194), width 135 (122–146) level with *JV2*. Ventrianal shield bearing several faint transverse lines, three pairs of simple spinose preanal setae *JV1–JV3* 26 (21–29), spinose *pan* 25 (22–28) and *pon* 18 (15–19), narrow cribrum and a pair of glands (*gv3*) on shield margin posterior of the anal opening. Ventral opisthosomal setae in soft integument simple and spinose, *ZV1* 17 (13–23), *ZV2* 25 (21–27) as long or nearly as long as *Jv* setae. Two pairs of glands (*gv2* and unknown paired-pore) and four pairs of poroids (*ivo*, *ivp*) in opisthosomal soft integument.

***Gnathosoma*** (Fig. 7). Basis capitulum medial length excluding internal malae 115 (113–116), width 139 (134–143) posterior to *pc.* Subcapitular setae simple: *h1* 44 (41–48), *h2* 16 (13–18), *h3* 57 (51–62), and *pc* 20 (19–23). Palp chaetotaxy normal for genus (2–5–6–14–15), palp apotele three-tined, *al* setae on trochanter, femur and genu slightly spatulate. Corniculi pointed, maximum length 39 (35–45), internal malae slender and smooth. Epistome tripartite with bifid central element bearing small fringe medially, lateral elements broad and flag-like distally, epistomatic margin finely serrate. Subcapitulum with seven rows: six rows have deutosternal denticles, the anterior most, and two posterior most rows with few (four) denticles laterally; the second anterior most row with paired ridges without any denticles. Chelicerae robust, length of second cheliceral segment including fixed digit 135 (122–141), and movable digit 49 (45–52). Fixed digit bidentate with one large and one small tooth, movable digit with a bidentate tooth flanked by a small tooth distally. Pilus dentilis and dorsal seta on fixed digit simple spikes, fixed digit with lyrifissure on each paraxial and antiaxial faces. Movable digit with narrow fringed arthrodial corona, and plumose arthrodial brush (47) almost as long as movable digit.

***Legs*** (Fig. 8). Excluding ambulacra, lengths of leg I 418 (409–432), leg II 390 (362–438), leg III 340 (319–358), and leg IV 474 (463–490). As in all *Macrocheles* ambulacra only present on legs II–IV, claws II–IV well developed. Pair of glands (*gc*) on coxa I. Setation of legs I–IV normal for Macrochelidae: coxae 2–2–2–1; trochanters 5–5–5–5; femora I (2–3/1,2/3–2), II (2–3/1,2/2–1), III (1–2/0,1/1–1), IV (1–2/1,1/0–1); genua I, II (2–3/1,2/1–2), III (1–2/1,2/0–1), IV (1–2/1,2/0–0); tibiae I (2–3/2,2/1–2), II (2–2/1,2/1–2), III, IV (1–1/1,2/1–1); tarsus I 20 setae plus numerous tapered setae dorsoterminally, tarsi II–IV 18. Most leg setae simple, setiform, femur II *ad1*, III *pd1*, IV *ad2*, *pd1*, and genu II *ad3* prominent spike setae with flattened forked tip with two to four tines that can appear as a single tapered point viewed laterally. Tarsus II with four large distal, and one ventral, spike setae with thickened conical base and rounded tip. Tarsi III, IV with four distal spike setae with wide base and flexible filamentous tip. Genu and tibia IV with slight ridge posterolateral.

###### Male and immatures.

Unknown.

###### Etymology.

*Pratum* (Latin neuter noun) means “meadow”. This species was only collected in Kearney County, Nebraska and Onefour, Alberta, which are in the prairies.

###### Remarks.

The female of *Macrocheles pratum* sp. n. is most similar to those of *M. willowae* sp. n., *M. nemerdarius*, *M. spinipes* Berlese, and *M. grossipes* Berlese. *Macrocheles pratum* sp. n. differs from *M. willowae* sp. n. as outlined in the *M. willowae* sp. n. description.

Female *M. pratum* sp. n. differs from that of *M. nemerdarius* in having larger more prominent punctures on the sternal shield, the posterior margin of the sternal shield is more concave, *pon* seta is smooth not weakly pilose, *j1* is only slightly longer than *z1* not 1.5 times as long, *J5* is shorter and broader for *M. pratum* sp. n. (9) than for *M. nemerdarius* (13), and the ventrianal shield is narrower, length to width ratio of 1.4 for *M. pratum* sp. n. and 1.2 for *M. nemerdarius*. Measurements were made examining *M. nemerdarius* holotype specimen loaned from the National Museum of Natural History, Smithsonian Institution.

*Macrocheles spinipes* and *M. grossipes* are associated with coprophilous beetles (Krantz 1988). Female *M. pratum* sp. n. differs from those of *M. spinipes* and *M. grossipes* in having larger more prominent punctures and transverse lines on the sternal shield, the posterior margin of the sternal shield more concave, arthrodial brush nearly as long as movable digit and not half or three quarters as long as movable digit, setae *ad2* and *pd1* on femur IV are large spike-like setae with flattened forked tips with two to four tines, ventrianal shield tapers relatively more towards posterior starting anterior of *pan* setae, and ventrianal shield is narrower, length to width ratio of 1.4 for *M. pratum* sp. n. and 1.2 for *M. spinipes* and *M. grossipes*. Measurements were made examining *M. spinipes* and *M. grossipes* voucher material from the Oregon State University Arthropod Collection.

##### Macrocheles kaiju sp. n.

Taxon classificationAnimaliaMesostigmataMacrochelidae

http://zoobank.org/1AFFAB07-D38F-429E-9F19-A76E2254787E

**Figure 9.**
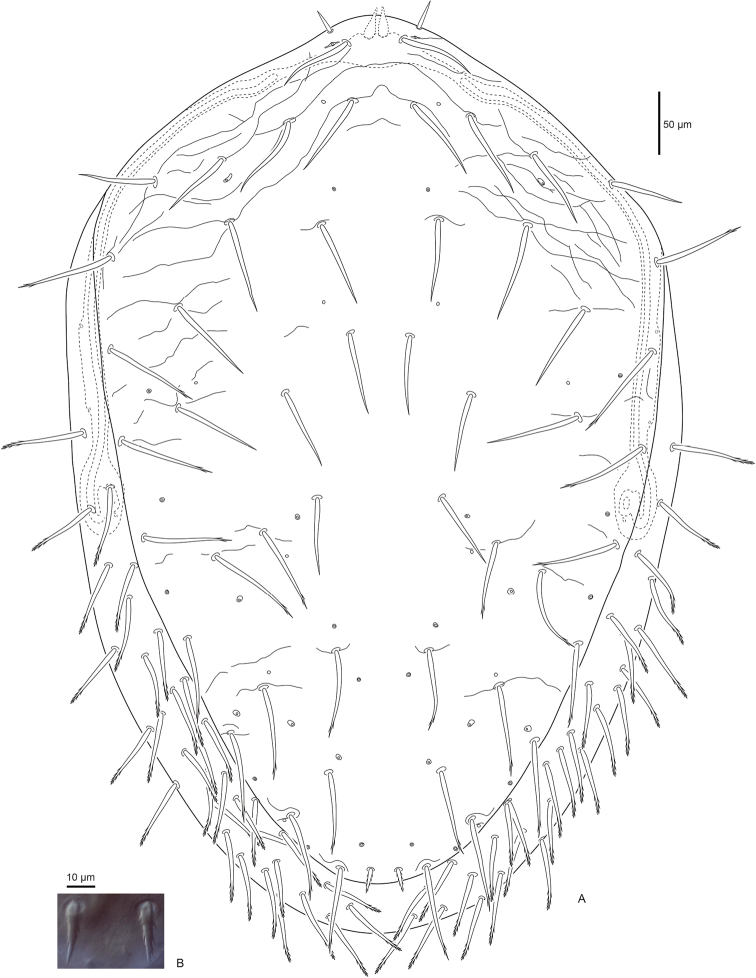
Female *Macrocheles kaiju* sp. n. **A** dorsal idiosoma **B** seta *J5*.

**Figure 10.**
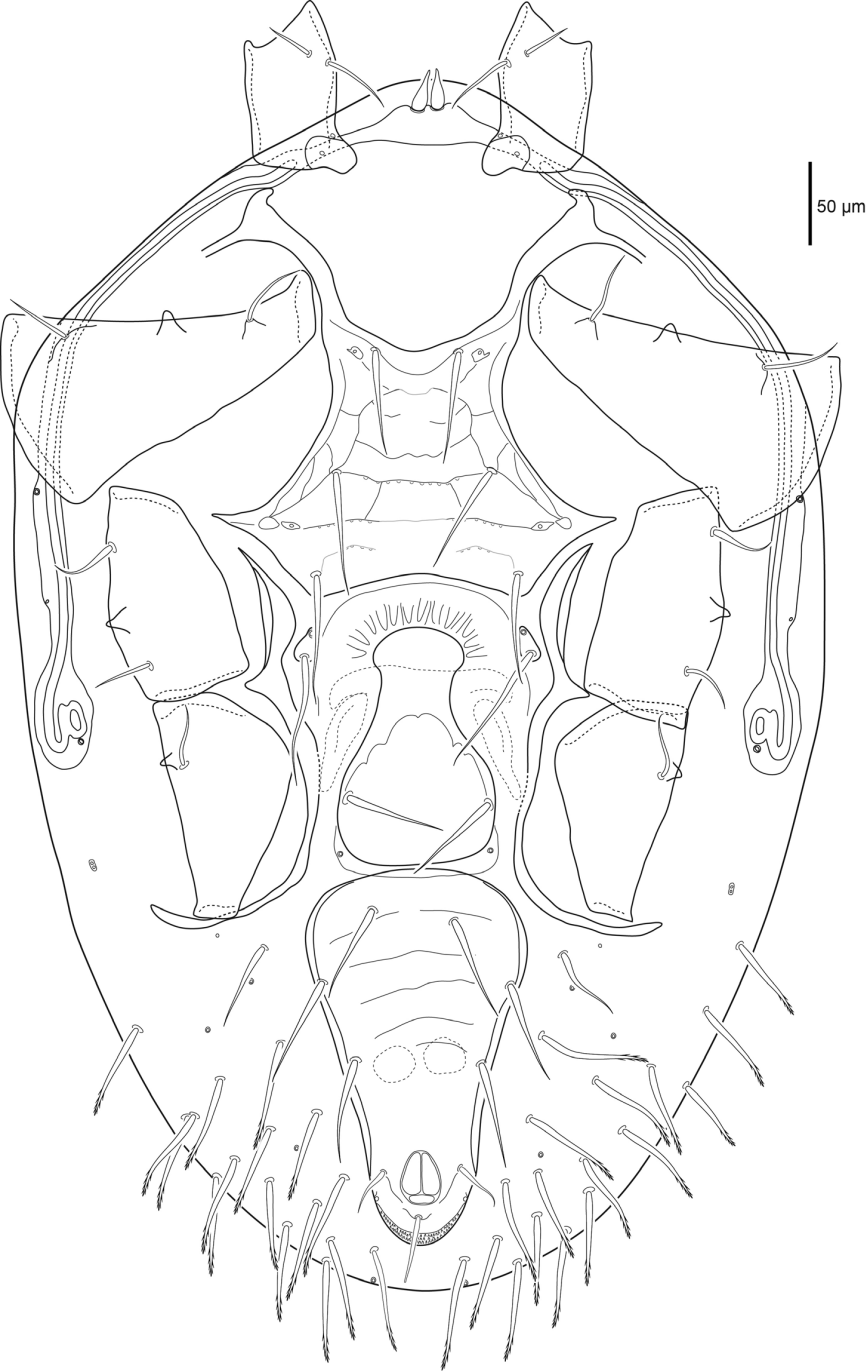
Female *Macrocheles kaiju* sp. n. ventral idiosoma including coxae.

**Figure 11.**
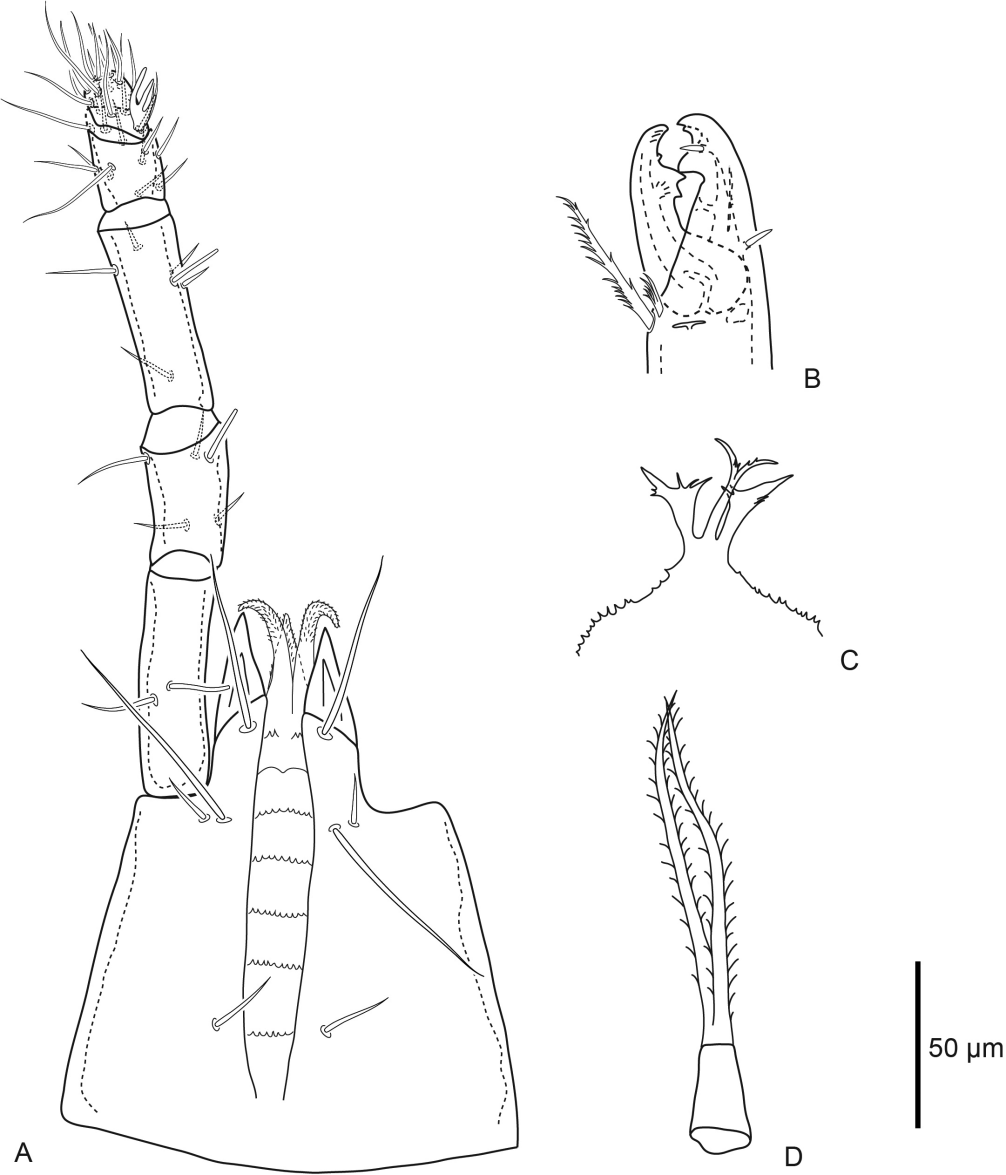
Female *Macrocheles kaiju* sp. n. **A** subcapitulum and palp, ventral aspect **B** chelicera, antiaxial aspect **C** epistome **D** tritosternum.

**Figure 12.**
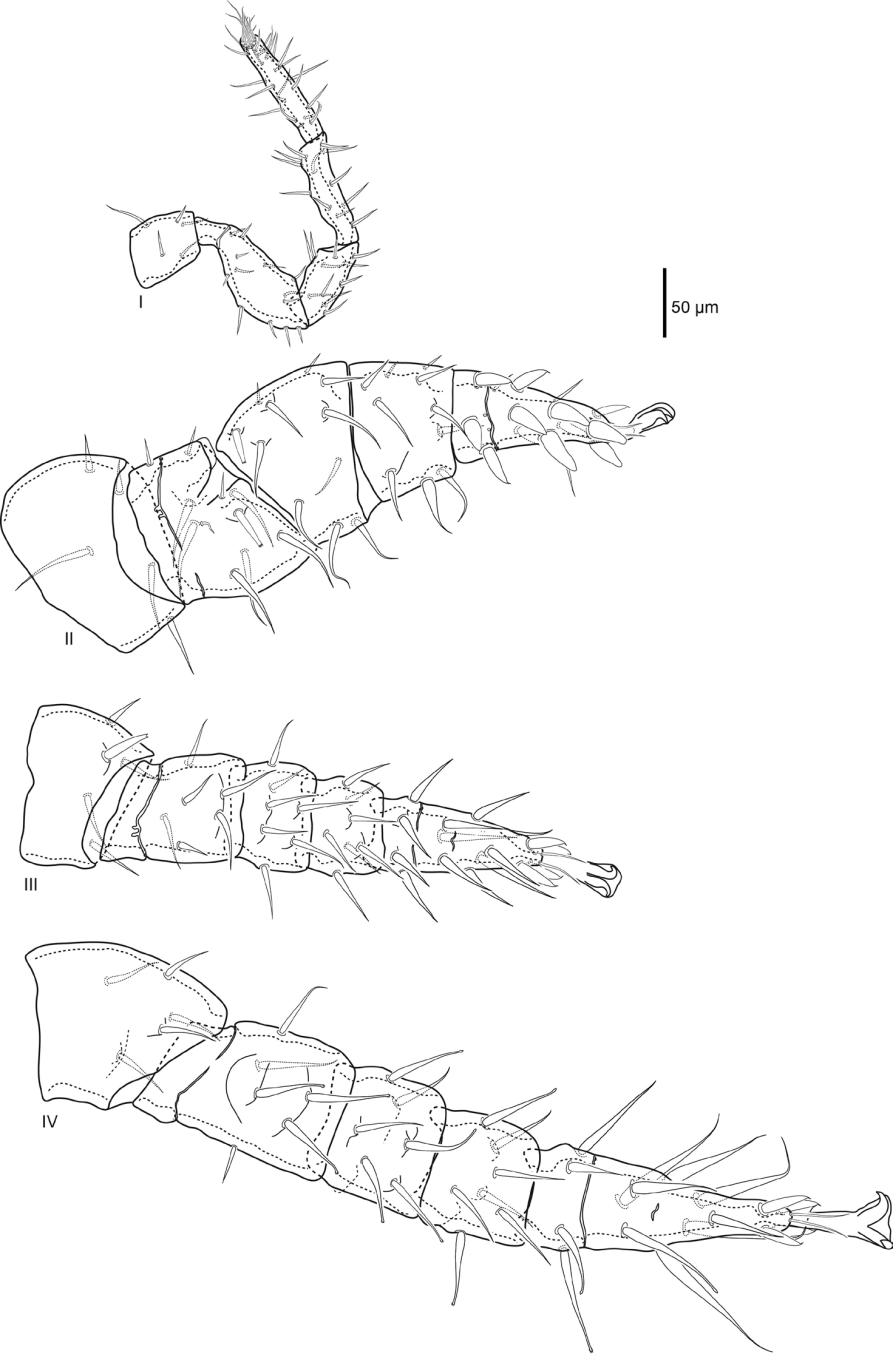
Female *Macrocheles kaiju* sp. n. legs I–IV, coxae omitted; leg I anterolateral, II posterolateral, III and IV dorsal.

**Figure 13.**
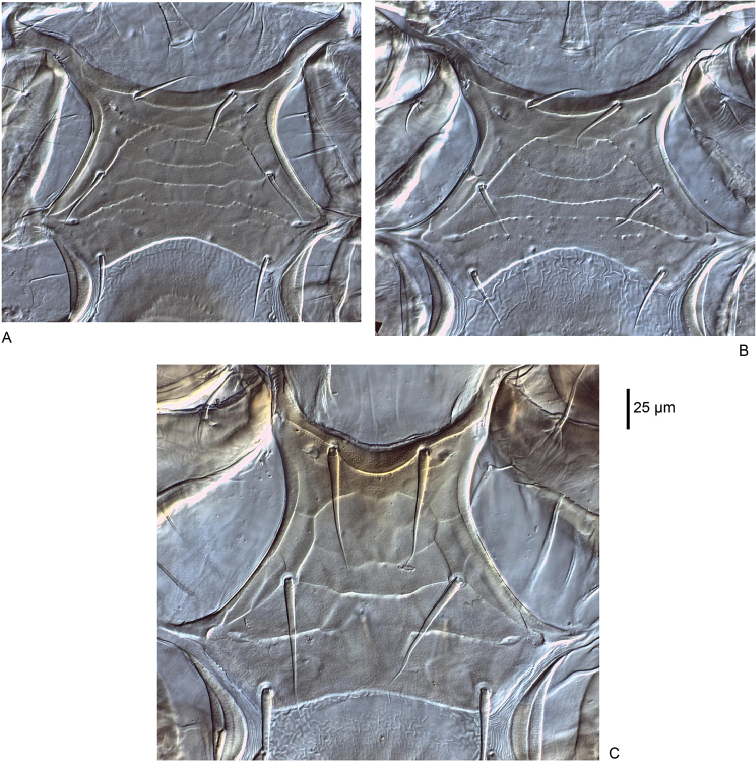
Sternal shield **A** *Macrocheles willowae* sp. n. **B** *M. pratum* sp. n. **C** *M. kaiju* sp. n.

Figs 9 , 10 , 11 , 12 , 13C

###### Material examined.

***Type material*.** Holotype: female (CNC829453) on *Nicrophorus carolinus* (N333, male) collected Highlands Co. Lake Placid, Florida, USA (27.181, -81.352), 10.iii.2009, coll: W. Knee & S. Peck.

Paratypes (28): 14 females (CNC CNC829454–829467) with the same collection information as the holotype; 13 females (CNC829468–829480) on *N. carolinus* (N334, female), Highlands Co. Lake Placid, Florida, USA (27.181, -81.352), 10.iii.2009, coll: W. Knee & S. Peck; female (CNC829481) on *N. carolinus* (N081), Highlands Co. Lake Placid, Florida, USA (27.181, -81.352), 10.iii.2009, coll: W. Knee & S. Peck.

***Other material*.** 134 mites examined from Florida and Nebraska on *N. carolinus* (Table 1).

###### Diagnosis female.

Dorsal setae smooth and spinose, except *r3*, *r4*, *s6*, *z6*, *S1*–*S5*, *Z1–Z5*, *J2* barbed distally, *J5* barbed, marginal and submarginal setae barbed distally. Seta *j1* smooth, spike, tapered distally with rounded tip, slightly longer than *z1*. Seta *J5* much shorter than *Z5*. Dorsal hexagonal setae as long as marginal and submarginal setae. Dorsal shield smooth medially with faint reticulations near shield margins, shield tapers from humeral region to posterior margin. Dorsal shield without well-defined procurved line, sigillary rami absent. Setae on sternal, genital and ventrianal shields, and *ZV1* smooth and spinose, other ventral setae in soft integument barbed distally. Sternal shield wider than long, punctures small, posterior margin slightly concave. Well defined l.m.t. and l.o.a.; l.o.a. contacts l.m.t. Well defined l.arc. contacts l.o.a., l.ang. and l.o.p. well defined laterally but faint medially. Well defined a.p.l., but a.p.p. not well defined. Ventrianal shield longer than wide (ratio 1.5), *pon* longer than *pan*, *pon* slightly spatulate. Arthrodial brush nearly as long as movable digit. Genu IV with six setae.

###### Description female.

***Dorsal idiosoma*** (Fig. 9). Dorsal shield 736 (647–812) long and 484 (421–547) (n=8) wide (level with *r3*), with 28 pairs of setae. Dorsal setae smooth and spinose, except *r3*, *r4*, *s6*, *z6*, *S1*–*S5*, *Z1–Z5*, *J2* barbed distally, *J5* barbed, marginal and submarginal setae barbed distally. Seta *J5* 22 (19–24) less than half as long as *Z5* 75 (65–83). Seta *j1* 29 (25–34) smooth, spike, tapered distally with rounded tip, slightly longer than *z1* 24 (20–32). Dorsal hexagonal setae 61 (50–71) smooth and spinose, as long as distally barbed marginal and submarginal setae (61). Dorsal shield smooth medially with faint reticulations near shield margins, shield tapers from humeral region to posterior margin. Dorsal shield without well-defined procurved line, sigillary rami absent. Dorsal shield fused to peritrematal shield near *r3* and anterior margin of shield wraps around onto ventral surface, *j1* on slight projection and typically on the venter, and *z1* occasionally expressed ventrally. Shield with 22 pairs of pore-like structures, of which six are secretory glands and 16 are non-secretory poroids.

***Ventral idiosoma*** (Figs 10, 13C). Sternal shield wider than long, medial length 155 (145–163), maximum width 260 (224–288) level with a.p.l., and minimum width 115 (103–121) posterior of *st1*. Sternal shield punctures small, posterior margin slightly concave. Setae *st1–3* 75 (61–92) simple and spinose, and two pairs of lyrifissures (*iv1*, *iv2*) on sternal shield. Pear-shaped metasternal shields well separated from sternal shield margin bearing lyrifissure *iv3* anteriorly and spinose seta *st4* 80 (73–89) posteriorly. Well defined l.m.t. and l.o.a.; l.o.a. contacts l.m.t. Well defined l.arc. contacts l.o.a., l.ang. and l.o.p. well defined laterally but faint medially. Well defined a.p.l., a.p.p. not well defined. Genital shield length 196 (172–226), width 123 (109–141) level with *st5*. Genital shield truncate posteriorly and hyaline margin rounded anteriorly, spinose seta *st5* 70 (65–74) on shield, pair of lyrifissures *iv5* off shield near posterior margin. Transverse line on genital shield well defined, and without punctures. Peritrematal shield narrow, fused to dorsal shield near *r3*, peritreme extends beyond posterior margin of coxa I, two poroids (*id3*, *id7*) and one gland (*gd3*) on the shield. Ventrianal shield longer than wide (ratio 1.5); length 230 (202–250), width 149 (135–160) anterior to *JV2*. Ventrianal shield bearing several faint transverse lines, three pairs of simple spinose preanal setae *JV1–JV3* 65 (64–75), spinose *pan* 37 (26–43) shorter than slightly spatulate *pon* 47 (40–54), narrow cribrum and a pair of glands (*gv3*) on shield margin posterior of the anal opening. Seta *ZV1* 55 (48–60) is simple, all other ventral opisthosomal setae in soft integument barbed distally, *ZV1* and *ZV2* 67 (58–75) as long or nearly as long as *Jv* setae. Two pairs of glands (*gv2* and unknown paired-pore) and four pairs of poroids (*ivo*, *ivp*) in opisthosomal soft integument.

***Gnathosoma*** (Fig. 11). Basis capitulum medial length excluding internal malae 159 (152–169), width 158 (153–170) posterior to *pc.* Subcapitular setae simple: *h1* 66 (61–70), *h2* 19 (17–21), *h3* 92 (85–98), and *pc* 25 (24–28). Palp chaetotaxy normal for genus (2–5–6–14–15), palp apotele three-tined, *al* setae on trochanter, femur and genu slightly spatulate. Corniculi pointed, maximum length 50 (45–60), internal malae thick and bristled. Epistome tripartite with bifid central element bearing small fringe medially, lateral elements broad and flag-like distally with irregular barbs, epistomatic margin finely serrate. Subcapitulum with seven rows, six of which have deutosternal denticles; the anterior most row with few (four) denticles laterally, and the second anterior most row with paired ridges without any denticles. Chelicerae robust, length of second cheliceral segment including fixed digit 184 (173–198), and movable digit 64 (60–67). Fixed digit bidentate with one large and one small tooth, movable digit with a bidentate tooth flanked by a small tooth distally. Pilus dentilis and dorsal seta on fixed digit simple spike, fixed digit with lyrifissure on each paraxial and antiaxial faces. Movable digit with narrow fringed arthrodial corona, and plumose arthrodial brush (57) almost as long as movable digit.

***Legs*** (Fig. 12). Excluding ambulacra, lengths of leg I 512 (488–527), leg II 576 (525–622), leg III 482 (436–528), and leg IV 690 (628–737). Ambulacra only present on legs II–IV, claws II–IV well developed. Slight ridge on femur II anterolateral, not always easily visible. Slight ridge on femur IV dorsal, genu and tibia IV posterolateral. Pair of glands (*gc*) on coxa I. Setation of legs I–IV normal for Macrochelidae: coxae 2–2–2–1; trochanters 5–5–5–5; femora I (2–3/1,2/3–2), II (2–3/1,2/2–1), III (1–2/0,1/1–1), IV (1–2/1,1/0–1); genua I, II (2–3/1,2/1–2), III (1–2/1,2/0–1), IV (1–2/1,2/0–0); tibiae I (2–3/2,2/1–2), II (2–2/1,2/1–2), III, IV (1–1/1,2/1–1); tarsus I 20 setae plus numerous tapered setae dorsoterminally, tarsi II–IV 18. Setae on leg I are setiform and simple, setae on legs II–IV variable, most are setiform, others are variously modified. Femur II *ad1*, genu II *ad3*, and trochanter III with a prominent spike setae with flattened forked tip with two to four tines that can appear as a single tapered point viewed laterally. Tarsus II with 13 thick conical spike setae with either a rounded or filamentous tip; filamentous tip fragile and easily broken. Tarsi III, IV with four distal spike setae with wide base and flexible filamentous tip, tip easily broken. Long setae with small spatulate tip with or without barbs on femur IV *ad1, ad2, pd1*, genu IV *al, ad1, pd1, pd2*, tibia IV *al, pl*, and three on tarsus IV. Setae with small spatulate tip appear pointed when viewed laterally.

###### Male and immatures

. Unknown.

###### Etymology.

Kaiju, 怪獣, from Japanese means strange beast, and refers to giant monsters such as Godzilla or Mothra. Female *M. kaiju* sp. n. is relatively unique morphologically when compared to other *Macrocheles* species associated with beetles, it is relatively large, has a unique dorsal shield shape, and bears numerous setae with distinct forms.

###### Remarks.

Female *M. kaiju* sp. n. is different from that of any other described *Macrocheles* species; however, it does fit the *Macrocheles* generic description (see Hyatt and Emberson 1988). Female *M. kaiju* sp. n. has two character states that are irregular for *Macrocheles* but somewhat similar to *Holostaspella* (Macrochelidae) species: the anterior margin of the dorsal shield wraps around onto the ventral surface such that *j1* is on a slight projection on the venter; and a slight ridge is present on femur II. *Holostaspella* species are characterised in part by females having a spur on femur II, and *j1* being on a tuberculate anterior extension of the dorsal shield but not wrapped around onto the venter. *Macrocheles kaiju* sp. n. differs from that of *Holostaspella* species in having weak ornamentation on the dorsal shield, its lateral regions without a series of depressions; *j1* is smooth and not pectinate, *j1* is on a slight projection and not a prominent tuberculate extension; sternal shield weakly ornamented and without strong median ridge; and metasternal shields small and always free of endopodal shields.

### Phylogenetics

COI was amplified from 14 *Macrocheles* specimens representing six species, with 689 characters in total, 430 constant, 39 parsimony-uninformative, 220 parsimony-informative. NJ analysis (K2P) was performed on 14 ingroup *Macrocheles* specimens. Average intraspecific pairwise distance was low (1.2% ±1.4), and the maximum intraspecific divergence observed was for *M. willowae* sp. n. (2.8%). The higher than average intraspecific divergence for *M. willowae* sp. n. was due to the divergence between mites from different host species. Pairwise distance between *M. willowae* sp. n. mites from *N. defodiens* and those from *N. orbicollis* and *N. carolinus* was higher than average (5.2% ±0.1), while divergence among mites from *N. defodiens* and mites from *N. orbicollis* and *N. carolinus* was low, 0.1 and 0.6% respectively. Mean interspecific divergence was high (20% ±2.8), and the maximum divergence observed was between *M. nataliae* and *M. subbadius* (25%). The range of intra- (0.3–2.8%) and interspecific (15–25%) pairwise divergence did not overlap.

**Figure 14.**
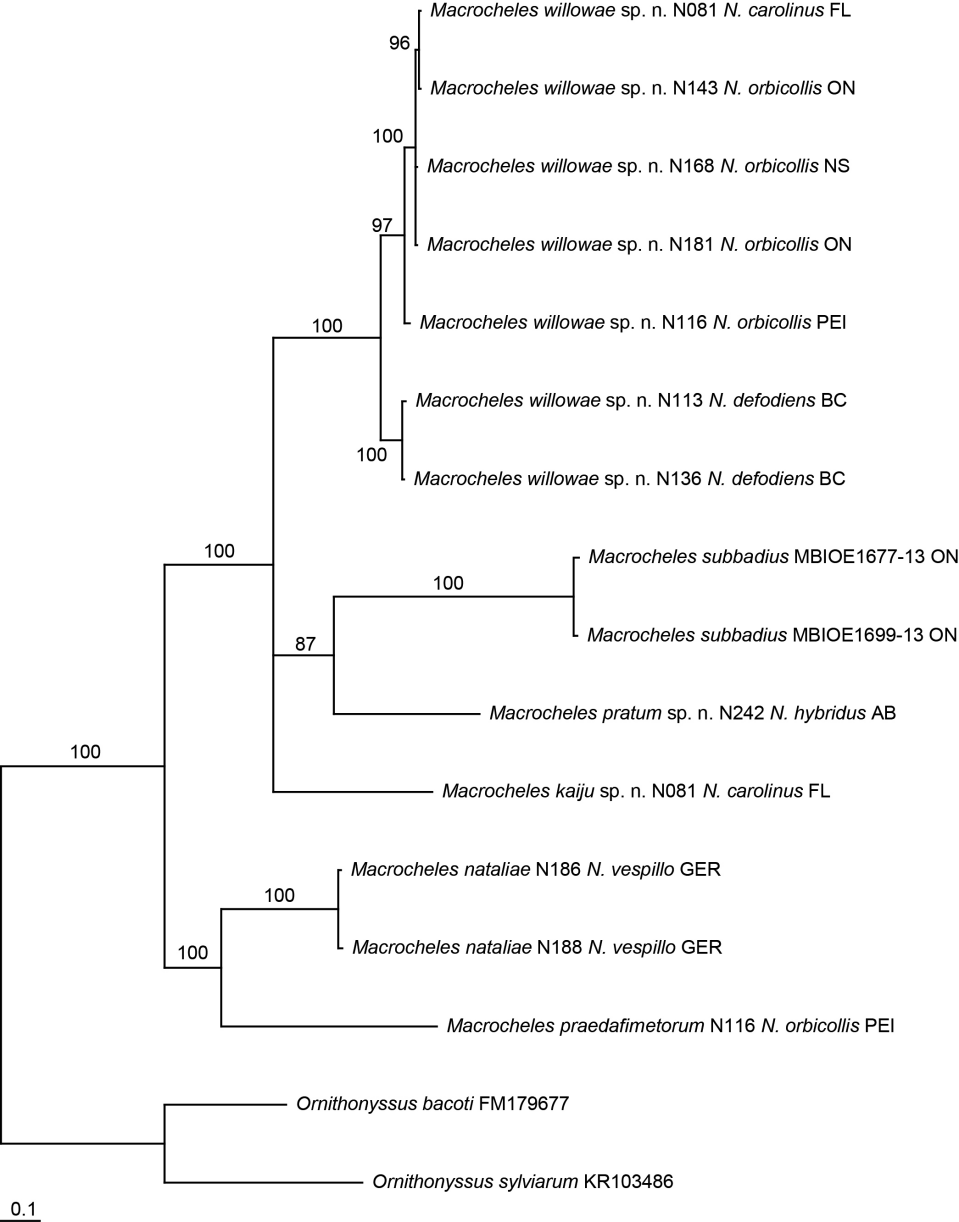
Majority rule consensus tree of 19502 trees generated by Bayesian MCMC analysis (10 million generations) of 689bp fragment of COI from 14 ingroup specimens representing six *Macrocheles* species, and two outgroup specimens representing two species, posterior probabilities >50% shown above branches.

The majority rule consensus tree from the BI analysis of COI was well supported, with all nodes having high posterior probabilities, and eight nodes with 100% support (Fig. 14). *Macrocheles willowae* sp. n. was divided into two well supported clades, one with mites from *N. defodiens* and the other with mites from *N. orbicollis* and *N. carolinus*. *Macrocheles willowae* sp. n. did not appear to diverge based on geographic location (Fig. 14). *Macrocheles willowae* sp. n. mites from *N. defodiens* and those from *N. orbicollis* and *N. carolinus* were morphologically indistinguishable, despite the higher than average intraspecific divergence between these two well supported clades. The phylogenetic relationships between *Macrocheles* species, and the genetic structure of these newly described species, requires further analysis with better taxon sampling, specimens from more host species and localities, and additional molecular markers.

### Distribution and biology

Seven species of macrochelids were collected from 112 *Nicrophorus* beetles representing nine species from three countries (Canada, USA, Germany) and 10 provinces or states (Table 1). *Nicrophorus carolinus* was associated with the most *Macrocheles* species (3), three host species had only two macrochelid species, and five had only one macrochelid species. Mites were usually found under the elytra, either clasping onto the integument near the prospiracle (68%) or on the ventral surface of the elytra (22%), and sometimes they were on the coxae (10%). Mites attached to the outer surface of the beetle could have been dislodged into the preservative.

A total of 1659 *Macrocheles* mites were collected from 112 beetles, *M. willowae* sp. n. (1268 mites on 86 beetles) was the most abundant, second most abundant was *M. pratum* sp. n. (196 mites on 15 beetles), *M. kaiju* sp. n. (163 mites on 7 beetles) was the third most abundant, and the four other species of *Macrocheles* collected were at low abundances (32 mites total on 10 beetles) (Table 1). *Macrocheles pratum* sp. n. was collected from five host species and had the greatest host range of all species collected. *Macrocheles willowae* sp. n. collected from three host species had the second broadest host range. *Macrocheles nataliae*, *M. praedafimetorum*, and *M. kaiju* sp. n. were each collected from a single host species.

The species with the greatest geographic range was *M. willowae* sp. n., collected from 22 sites, across seven provinces/states in Canada and USA. *Macrocheles pratum* sp. n. was collected from a single site in Alberta (Canada) and from another site in Nebraska (USA). *Macrocheles kaiju* sp. n. was collected from one site in Florida and another site in Nebraska, USA. The four other macrochelid species collected were each found in a single locale (Table 1).

COI sequences generated in this study were compared against those on GenBank and BOLD, and *M. willowae* sp. n. was the only species to have high level (100%) matches on BOLD. These matching sequences belonged to generic level identified specimens from Alberta, Ontario, Saskatchewan, Nova Scotia, and Florida. The species briefly diagnosed by Dr. W. Yoder was collected from three species of rodents in Maryland, Michigan and Prince Edward Island. Combined together, results from this study, BOLD and Dr. W. Yoder’s findings, the geographic distribution of *M. willowae* sp. n. may cover 11 provinces or states in Canada and USA.

## Supplementary Material

XML Treatment for Macrocheles

XML Treatment for Macrocheles willowae

XML Treatment for Macrocheles pratum

XML Treatment for Macrocheles kaiju
